# Whole genome sequence analysis reveals genetic structure and X-chromosome haplotype structure in indigenous Chinese pigs

**DOI:** 10.1038/s41598-020-66061-2

**Published:** 2020-06-10

**Authors:** Xiong Tong, Lianjie Hou, Weiming He, Chugang Mei, Bo Huang, Chi Zhang, Chingyuan Hu, Chong Wang

**Affiliations:** 1 National Engineering Research Center for Breeding Swine Industry, Guangdong Provincial Key Lab of Agro-Animal Genomics and Molecular Breeding, College of Animal Science, South China Agricultural University, Guangzhou, Guangdong 510642 China; 2 State Key Laboratory of Agricultural Genomics, BGI Genomics, BGI-Shenzhen, Shenzhen, 518083 China; 3 State Key Laboratory of Livestock and Poultry Breeding, Institute of Animal Science, Guangdong Academy of Agricultural Sciences, Guangzhou, 510640 China; 4 College of Animal Science and Technology, Northwest A&F University, Yangling, 712100 China; 5 Department of Human Nutrition, Food and Animal Sciences, University of Hawaii at Manoa, 1955 East-West Road, AgSci. 415J, Honolulu, HI 96822 USA

**Keywords:** Rare variants, Genetic markers

## Abstract

Chinese indigenous pigs exhibit considerable phenotypic diversity, but their population structure and the genetic basis of agriculturally important traits need further exploration. Here, we sequenced the whole genomes of 24 individual pigs representing 22 breeds distributed throughout China. For comparison with European and commercial breeds (one pig per breed), we included seven published pig genomes with our new genomes for analyses. Our results showed that breeds grouped together based on morphological classifications are not necessarily more genetically similar to each other than to breeds from other groups. We found that genetic material from European pigs likely introgressed into five Chinese breeds. We have identified two new subpopulations of domestic pigs that encompass morphology-based criteria in China. The Southern Chinese subpopulation comprises the classical *South Chinese Type* and part of the *Central China Type*. In contrast, the Northern Chinese subpopulation comprises the *North China Type*, the *Lower Yangtze River Basin Type*, the *Southwest Type*, the *Plateau Type*, and the remainder of the *Central China Type*. Eight haplotypes and two recombination sites were identified within a conserved 40.09 Mb linkage-disequilibrium (LD) block on the X chromosome. Potential candidate genes (*LEPR*, *FANCC*, *COL1A1*, and *PCCA*) influencing body size were identified. Our findings provide insights into the phylogeny of Chinese indigenous pig breeds and benefit gene mining efforts to improve major economic traits.

## Introduction

Approximately 10,000 years ago, pigs *(Sus scrofa* L.) were independently domesticated in multiple Eurasian regions^1,2^. China is a major center of early pig domestication^3^ and therefore has numerous indigenous breeds that exhibit considerable phenotypic variation in response to both artificial and natural selection. Except for wild boars, Chinese indigenous pigs are historically classified into 48 breeds and split into six types (*South Chinese*, *North China*, *Lower Yangtze River Basin*, *Central China*, *Southwest*, and *Plateau*), based on geographic distribution, historical origin, and morphological characteristics^4^. Some molecular evidences^5–8^ suggests that this classification may be problematic, given the potential for admixture among different types. However, these studies used a small number of molecular markers, including randomly amplified polymorphic DNA^5^ and microsatellites^6–8^, and therefore this admixture has not been well studied yet.

With the development of genome sequencing and SNP chip technologies, the past decade has seen an increase in data on genome-wide variation. Indeed, comparative genomic analyses have identified genes involved in a wide range of agriculturally-important traits, including coat color^9,10^, body size^11–13^, meat yield^11^, and disease resistance^11^. DNA-based techniques provide an excellent opportunity to clarify the Chinese pig classification. Recent studies investigated only a few breeds with highly desirable production-related traits^10,13^, and focused on identifying selective sweeps during domestication^14,15^. Such research included genome-wide analyses of domestic breeds (e.g., Tibetan^14^, Tongcheng^10^, Enshi Black^13^, and Rongchang^16^) with a focus on tolerance to harsh environments, high fertility, and body size. Currently, too few Chinese pig breeds have been studied to provide a conclusive investigation of porcine evolution in China. Specific loci and genes underlying common phenotypic variation among Chinese domestic pig breeds have not yet been studied.

To address these deficiencies, we performed whole-genome resequencing of pigs representing 22 breeds distributed across different geographical areas in China. This new sequence data was integrated with publically-available sequence data from seven other pig breeds, including European breeds. We uncovered population genetic structures among Chinese indigenous pigs, genetic introgression between population pairs (North China, South China, and Europe), LD patterns of X-chromosome, along with potential candidate genes associated with body size.

## Results and discussion

### Sequencing and variation identification

Twenty-four animals representing twenty-two pig breeds were individually resequenced (Table 1 and Supplementary Fig. S1 and Table S1). The average effective sequencing depth was 17.54 (±7.30)×, and genomic coverage was 94.74 (±0.69)% (Supplementary Fig. S2 and Table S2).

**Table 1:** Summary of the sample information.

Classification Types	Abbreviation	Sex	Breed	Area of Origin	Latitude, longitude, altitude (m)
*South Chinese* (n = 10)	BM	M	Bama Xiang	Bama Yao Autonomous County, Guangxi province, China	24.14°N, 107.26°E, 244 m
	BN	F	Banna	Dai Autonomous Prefecture of Xishuangbanna, Yunnan Province, China	22.01°N, 100.80°E, 1000 m
	DS	F	Diannan small-ear	Dai Autonomous Prefecture of Xishuangbanna, Yunnan Province, China	22.01°N, 100.80°E, 1000 m
	HX	M	Huanjiang Xiang	Huanjing Maonan Ethnic Autonomous County, Guangxi province, China	24.83°N, 108.26°E, 740 m
	LC	M	Luchuan	Luchuan County, Guangxi Province, China	22.25°N, 110.26°E, 100 m
	LL	M	Longlin	Multinational Autonomous County of Longlin, Guangxi Province, China	24.77°N, 105.34°E, 1826m
	LT	M	Lantang	Zijin County, Guangdong Province, China	23.41°N, 11494 °E, 300 m
	WZSI	M	Wuzhi Shan (inbreeding)	Institute of Animal Sciences, Chinese Academy of Agricultural Sciences, Beijing, China.	
	WZSO	M	Wuzhi Shan (origin)	Five Fingers Group City, Hainan Province, China	18.78°N, 109.51°E, 325 m
	YH	M	Yuedong Hei	Huiyang District, Guangdong province, China	22.79°N, 114.45°E, 31 m
*Central China* (n = 3)	DH	M	Dahua Bai	Xingning City, Guangdong Province, China	24.14 °N, 115.73 °E, 50 m
	JH	M	Jinghua	Jinhua City, Zhejiang Province, China	29.08 °N, 119.64 °E, 43 m
	NX	M	Ningxiang	Ningxiang County, Hunan Province, China	28.28 °N, 112.55 °E, 120 m
*North China* (n = 2)	LWU	M	Laiwu	Laiwu City, Shandong Province, China	36.21°N, 117.67 °E, 994 m
	MP	M	Min	Three provinces in Northeast China	43.89°N, 125.32 °E, 240 m
*Lower Yangtze River Basin* (n = 2)	EHL	M	Erhua Lian	Wuxi City, Jiangsu Province, China	31.49°N, 120.31 °E, 10 m
	MS* (ERR173202)	NA^#^	Meishan	Taicang City, Jiangsu Province, China	31.46°N, 121.13 °E, 3 m
*Southwest* (n = 1)	NJ	M	Neijiang	Neijiang city, Sichuan province, China	29.58°N, 105.06 °E, 450 m
*Plateau* (n = 2)	GZT	M	Ganzi Tibet	Ganzi Tibetan autonomous prefecture, Sichuan province, China	30.05°N, 100.30°E, 3200 m
	HZT	M	Hezuo Tibet	Gannan Tibetan autonomous prefecture, Gansu province, China	34.98°N, 102.91°E, 2,881 m
European domestic breeds (n = 5)	DP	F	Duroc	Denmark, North American	56.26°N, 9.50°E, 51 m
	HP* (ERR173174)	NA^#^	Hampshire	England	52.36°N, -1.17°E, 104 m
	LP	F	Landrace	Denmark, North American	56.26°N, 9.50°E, 51 m
	LW	F	Large white	England	52.36°N, -1.17°E, 104 m
	PP* (ERR173208)	NA^#^	Pietrain	Belgium	50.50°N, 4.47°E, 140 m
Wild boar (n = 6)	IW* (ERR173218)	NA^#^	Switzerland	Malcantone region, Switzerland	46.05°N, 8.90°E, 810 m
	NCW1	M	North China1	Three provinces in Northeast China	47.12°N, 128.74°E, 386 m
	NCW2* (ERR173222)	NA^#^	North China2	Three provinces in Northeast China	43.15°N, 126.44°E, 683 m
	NW* (ERR173214)	NA^#^	Netherlands	Veluwe region, Netherlands	52.13°N, 5.29°E, 37 m
	SCW1	M	South China1	Hainan Province, China	19.57°N, 109.95°E, 44 m
	SCW2* (ERR173220)	NA^#^	South China2	Yunnan Province, China	24.48°N, 101.34°E, 1646m

Notes: (1) *Downloaded data of seven individuals (one pig per breed) from the Wageningen University Porcine re-sequencing Phase 1 Project (https://www.ebi.ac.uk/ena/data/view/PRJEB1683).

(2) # NA represents null.

To these data, we included genomic data^12,17^ publically available for seven pigs of wild and commercial European and Chinese breeds (Table 1). The combined dataset had 14.09 billion high-quality raw reads (1,281.12 Gb raw bases, >90% Q30 bases) (Supplementary Fig. S3).

A strict quality-filter pipeline resulted in 19,685,697 single-nucleotide polymorphisms (SNPs) from 31 pigs (Supplementary Table S3). Of these SNPs, 13,430,360 (68.22%) were in intergenic regions, 1,223,834 (6.22%) were 5-Kb upstream or downstream of gene regions, and 5,031,503 (25.56%) were within gene regions. The last group contained 46,618 non-synonymous (NS) and 53,028 synonymous (S) SNPs (Supplementary Fig. S4), leading to an NS/S ratio (ω) of 0.88, which is higher than the ratio of 0.68 reported by Li *et al*.^14^. This study collected more local pig breeds in China than Li *et al*.^14^, resulting in a higher NS/S ratio. In this study, 20 Chinese domestic pig breeds covering the whole country were collected. In the study of Li *et al*.^14^, although the number of individuals reached 45, only Tibetan pigs and five other local Chinese breeds distributed in Sichuan and Chongqing were collected.

In addition, we identified 5,081,752 small-to-medium (1–20 bp) indels (Supplementary Table S4). As expected, most indels (3,486,145, 68.60%) occurred in intergenic regions; the remainder were either 5 Kb upstream or downstream of gene regions (352,227, 6.93%), or in gene regions (1,243,380, 24.47%). The Frameshift/Non-frameshift ratio was 2.24 (Supplementary Fig. S5). Larger structural variations (SV, >45 bp) were detected using read-pair and read-depth methods. Across individuals, the SV count varied from 2,881 to 49,939. Deletions and intra-chromosomal translocations were the two primary SV types identified in our samples (Supplementary Table S5).

Homozygous (Hom) and heterozygous (Het) SNPs were classified per individual. Homozygous SNPs were more common in all European pigs than in Chinese pigs, especially in two European wild boars that had Hom/Het SNP ratios of 3.804–4.460 (Supplementary Table S3). Furthermore, except for the Large White (LW) pig, higher Hom/Het ratios of indels were observed in European pigs than in Chinese pigs, which was consistent with that of SNP variants (Supplementary Table S4). These results suggest that population bottlenecks may be responsible for the reduced genetic diversity observed in European pigs compared with Chinese pigs^17^. Additionally, numerous specific alleles appear to have been fixed in European and Chinese populations after separation.

### Population structure and introgression

**Figure 1:**
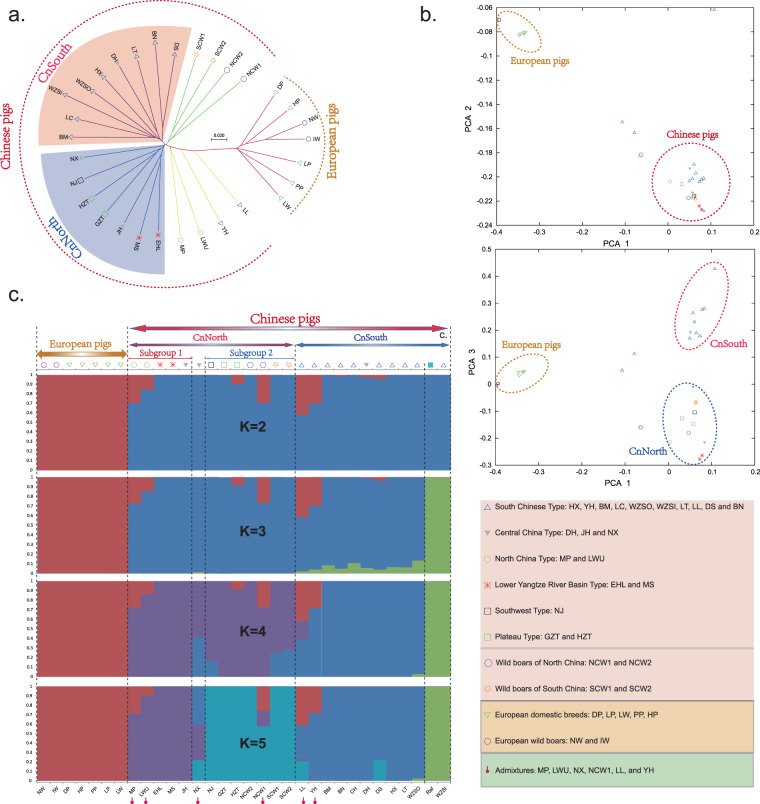
Population structure of wild and domestic pigs from different geographical regions. **(a**) Neighbor-joining tree of all pigs based on the 9.2 million population SNPs. The scale bar denotes *p* distance between individuals. **(b)** PCA1-2 and PCA1-3 plots of all individuals. **(c**) Genetic structure analysis of samples using FRAPPE, with changing ancestral populations from K = 2 to K = 5.

We constructed a non-rooted phylogenetic tree based on 9.2 million population SNPs (Fig. 1a and Supplementary Fig. S6) to understand the genetic relationships and structure among Chinese pigs with different geographical distributions. The estimated phylogeny revealed that the primary division was between European and Chinese pigs, European wild boars clustered with European domestic pigs, and Chinese wild boars clustered with Chinese domestic pigs, consistent with previous studies^14,17^. Our results lend further support to the viewpoint that pig domestication occurred independently in western Eurasia and East Asia. Moreover, Chinese domestic breeds split on geographical grounds, namely into South and North China (CnSouth and CnNorth) subpopulations. The former encompassed all individuals from the classical *South Chinese Type* and some of the *Central China Type*. The latter comprised the remainder of *Central China Type* and all those from the remaining four types (*North China*, *Lower Yangtze River Basin*, *Southwest*, and *Plateau*) (Fig. 1a). The genetic relationships among Chinese indigenous pig breeds were remarkably congruent with geographic distribution. Dahua Bai (DH: Xingning City, Guangdong Province, South China) clustered with *South Chinese Type* breeds and Jinhua (JH: Jinhua City, Zhejiang Province, Yangtze River lower reaches) clustered with *Lower Yangtze River Basin Type* breeds (Fig. 1a and Table 1). Notably, DH and JH are considered to be of the *Central China Type*, a consideration based on coat color phenotypes^4^. The reference genome selected in this study was also from inbred Wuzhishan pig, which belonged to the same inbred population as WZSI used in this study. After nearly 20 generations of inbreeding, the inbred line has formed distinct genetic differentiation with other local Chinese pigs, leading to a separate cluster, including the reference genome sample and WZSI at K = 3 (Fig. 1c).

Principle component analysis (PCA) confirmed the phylogenetic analysis (Fig. 1b and Supplementary Table S6). Furthermore, a model-based clustering analysis with proportional contributions from five ancestral populations revealed the same subpopulations (CnNorth and CnSouth). Northern Chinese pigs could be further split into two subgroups (Fig. 1c): Subgroup 1 consisted of the *Lower Yangtze River Basin* and *North China* types, and Subgroup 2 comprised the *Southwest* and *Plateau* types. Features of genetic structure (Fig. 1c) and geographical distribution (Supplementary Fig. S1) confirmed the three East-Asian centers of pig domestication identified initially through mitochondrial DNA. These centers are the Mekong region^18^, middle and downstream regions of the Yangtze River^19,20^, and Tibetan highlands^18,20^. Thus, our study provides evidence that the classical classification scheme^4,21^ requires updating with genetic information.

Our three analyses of population structure (phylogeny, PCA, and clustering analysis) (Fig. 1a–c) revealed that admixture likely took place in six Chinese indigenous breeds. Therefore, we employed the haplotype sharing ratio to examine putative introgression across all pairs of four populations (South China, North China, Europe, and admixed, including domestic and wild pigs) corresponding to our model-based clusters (Fig. 1c). All autosomes from South China, North China, and Europe populations contained numerous discrete introgression fragments, indicating extensive gene flow had occurred under artificial or natural evolutionary processes. Multiple large and dense regions on chromosomes 5, 14, 17, and 18 were introgressed from the European population into five Chinese breeds (Supplementary Fig. S7a–d). Similar events have been reported for Longlin^22^, Yuedonghei^22^, Min^23^, Kele^23^, and Zang/Tibetan^14^ breeds.

We examined nucleotide variation (θπ and θw) to measure genetic diversity across three populations (wild pigs, European domestic pigs, and Chinese domestic pigs) and the two Chinese subpopulations (CnNorth and CnSouth). Tested populations were more genetically-diverse (θw/Kb: 2.01–2.80, θπ/Kb: 2.12–3.11; Supplementary Table S7) than cattle breeds Angus and Holstein^24^ (θw/Kb and θπ/Kb: ~1.4), dogs^25^ (θw/Kb: 0.61–1.28, θπ/Kb: 0.75–1.38), and giant pandas^26^ (θw/Kb: 1.04–1.30, θπ/Kb: 1.13–1.37). In comparison with wild and Chinese domestic pigs, European domestic pigs have a lower level of genetic diversity (θw/Kb:2.01, θπ/Kb: 2.12). We then calculated the divergence index (*F*_ST_) to measure population differentiation between the different domestic pigs and wild pigs and between the two subpopulations (Supplementary Fig. S8). The highest *F*_ST_ (0.08) was observed between European domestic pigs and wild pigs. The LD decay rate was measured by the average distance over which the LD coefficient (*r*^2^) falls to half of its maximum value (Supplementary Fig. S9). The LD decay rate of European domestic pigs (~27.60 kb, *r*^2^_0.5_ = 0.33) was lower than that of the other two populations (wild pigs: ~7.30 kb, *r*^2^_0.5_ = 0.25; and Chinese domestic pigs: ~6.00 kb, *r*^2^_0.5_ = 0.27), which might be a result of the low genetic diversity in European domestic pigs. Taken together, our results from genetic diversity and LD decay in European domestic pigs support the hypotheses of expansion from a relatively small ancestral population^14,17^ and a large reduction of effective population size under intensive breeding^27^.

The bottleneck effect can greatly change the allele frequency of sites in the population, which is the main reason for the drastic change of LD in a short time^28^. In our study, within a short LD decayed distance (<30 Kb), wild pigs had lower *r*^*2*^ than Chinese pigs. However, higher *r*^2^ at a longer distance (≥30 Kb), suggests that the ancestral population from wild boars was larger than that from Chinese domestic pigs, but wild boars were subjected to narrow bottlenecks. The similar signatures of narrow bottlenecks within LD patterns have also been reported from different cattle populations^24^. Finally, CnNorth and CnSouth exhibited low population differentiation (*F*_ST_ = 0.06) and similar nucleotide diversity and LD decay rate (Supplementary Table S7 and Figs. S8 and S9b).

### Characterization of a large-scale LD block in the X chromosome

**Figure 2:**
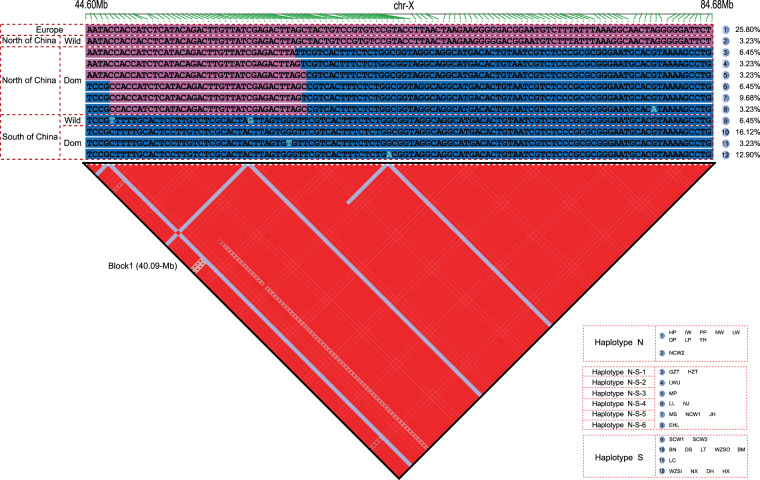
Haplotype pattern of LD block region on the chromosome X (1 SNP/0.3 Mb). Purple and blue represent the same or opposite alleles in the Wuzhishan reference genome, respectively. The percentages on the right are the proportions of samples with corresponding haplotypes in the total sample (n = 31). Red blocks represent LD blocks in the 40.09 Mb region. Haplotype S is identified in South China (domestic pigs and wild boars) (blue regions). Haplotype N is identified in European (domestic pigs and wild boars) as well as wild boar of North China (purple regions). Six derived Haplotypes (Haplotype N-S-1-6) are identified in domestic pigs of North China (purple and blue regions).

Using SNP data, we identified a large-scale LD block (40.09 Mb, 44,595,487–84,684,295 bp) (Fig. 2) in the X chromosomes of all 31 pigs. This region was previously shown to have an extremely low recombination rate (48 Mb segment, 44.0–91.5 Mb)^15,29^, and spanned the centromeric region (47.3–49.2 Mb). We observed three major haplotypes after selecting SNP markers with inter-marker distances of 3 Kb. Haplotype S was unique to domestic and wild pigs of southern China, whereas N was present in northern Chinese wild pigs, European domestic pigs, and European wild pigs. The third was a recombinant haplotype set that included six haplotypes (N-S-1 to N-S-6) found only in northern Chinese domestic pigs (Fig. 2). These LD patterns indicate that northern Chinese domestic pigs exhibit more haplotype diversity and they corroborate previous findings of a 14 Mb X-linked sweep region^12,15^.

**Figure 3:**
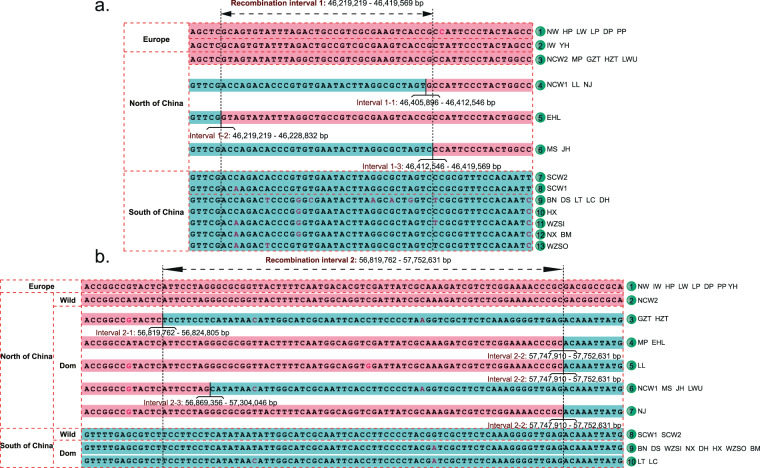
Physical location ranges of recombination interval 1 (**a**) and interval 2 (**b**) (The analysis used all SNPs in the LD block region). Red and blue represent the same or opposite alleles in the Wuzhishan reference genome, respectively. (**a**) Three types of recombination interval 1 (Interval 1-1, Interval 1-2, Interval 1-3) are identified in the domestic pigs of North China. The maximum range of recombination interval 1 is 46,219,219-46,419,569 bp. **(b)** Three types of recombination interval 2 (Interval 2-1, Interval 2-2, Interval 2-3) are identified in the domestic pigs of North China. The maximum range of recombination interval 2 is 56,819,762-57,752,631 bp.

We then used all SNP markers from the LD block to detect intervals of local breakdown in LD in the haplotype set. We identified two intervals of reduced recombination: interval 1 (left) at 46, 219, 219–46, 419, 569 bp and interval 2 (right) at 56, 819, 762–57, 752, 631 bp. The minimum distance between the two intervals was a 10.40 Mb segment (46, 419, 569–56, 819, 762 bp) (Fig. 3), a highly conserved portion of haplotype N in northern Chinese domestic pigs. Moreover, the 10.40 Mb segment is located inside the 14 Mb X-linked sweep^15^. Overall, we found more haplotypes (n = 8) within the 40.09 Mb LD block and a shorter conserved region (10.40 Mb) than described in the previous reports^12,15,29^, which were likely due to our use of high-density genetic markers from data with high sequencing depths and from obtaining a greater number of Chinese pig breeds.

The 40.09 Mb LD block contained 189 annotated genes, 143 (75.66%) and 108 (57.14%) of which contained SNPs and nonsynonymous substitutions, respectively. KEGG analysis mapped these 189 genes onto the Shigellosis and Neurotrophin-signaling pathway (Supplementary Tables S8 and S9). Of the 374X-chromosome QTLs in the Pig Quantitative Trait Locus database (Pig QTLdb), we aligned 247 (66.04%) to the Wuzhishan pig genome. Furthermore, 47X-chromosome QTLs overlapped with the 40.09 Mb LD block. Thirty-seven (37/47, 78.72%) and seven (7/47, 14.89%) QTLs were related to meat and carcass quality and reproduction, respectively (Supplementary Table S10). Within the meat and carcass quality associated QTLs, 26 (26/37, 70.27%) were related to fat traits (3 fat composition and 23 fatness QTLs), consistent with lipid-metabolism QTLs identified near the X-chromosome centromere^30^. Trait hierarchies for reproduction associated QTLs from the Pig QTLdb are divided into four categories: endocrine, litter traits, reproductive organs, and reproductive traits. In this study, the seven overlapping QTLs associated with reproduction traits were assigned to the reproductive organs, reflecting between-subpopulation (CnNorth, CnSouth, and European) differences in reproductive characters.

Across CnNorth and CnSouth pigs, we identified 4,169 population-level indels in CDS regions of functional genes. After filtering out markers that covered samples less than 5 in one group to meet the minimum requirement of an expected value of chi-square statistics, 2,711 indels remained. Six differed significantly between the two subpopulations, and five of these were distributed in three gene loci (ENSSSCG00000012830, HUWE1, and ITIH5L) in the 10.40 Mb conserved region (Supplementary Table S11). The first locus contained three indels that were matched against the InterPro database to reveal two specific cold-shock protein domains (IPR002059 and IPR011129). Variants of these genes in the CnNorth pigs were also found in northern Chinese wild pigs and European domestic and wild pigs.

We next selected the top 100 SVs out of 64,876 population-level SVs that exhibited significantly non-random distribution (χ^2^ test with *FDR* correction, *P* < 0.01). Thirty-four of these SVs were located in the X chromosome (Supplementary Table S12), with 32 in the 10.40 Mb conserved region. The conserved region contained 63 annotated genes, and four (*EDA*, *HEPH*, *ARHGEF9*, and *HUWE1*) overlapped with six SVs that exhibited very high between-group differences (*P* = 8.53 × 10^-4^) (Supplementary Table S13). We identified two large loss-of-function deletion patterns (382 bp: 56,650,381–56,649,999, and 487 bp: 56,621,617–56,621,130, Supplementary Table S13) on *EDA* and found that they were fixed only in CnNorth pigs. The EDA signaling pathway is involved in ectodermal-organ (hair, teeth, and exocrine glands) development^31,32^, and EDA defects result in Tooth Agenesis^32^. Our findings are consistent with archaeological evidence of different tooth structural characters between CnNorth and CnSouth pigs^4^.

### Identification of candidate genes for body size

Our sample was split into small pigs (adult body length ≤100 cm, height ≤50 cm; N = 7) and large pigs (adult body length ≥120 cm, height ≥65 cm; N = 7), based on early phenotype characterization records^21^ and our own measurements (Supplementary Table S14). We then identified 115 nonsynonymous substitutions, distributed in 95 gene regions, that differed in allele frequency between large versus small pigs (>80% in one group, approaching fixation; <20% in the other) (Supplementary Table S15). These nonsynonymous substitutions were putative candidate polymorphisms that resulted in size differences. Indeed, two genes (*LEPR* and *FANCC*) overlapping with nonsynonymous substitutions are reported as associated with body growth and development in some mammals^33,34^. In humans, impaired *LEPR* function exerts a strong negative effect on ponderal index at birth and height in adolescence^34^. Likewise, *FANCC* plays a major role in skeletal formation, and thus affects human height^35,36^.

We then analyzed differences (χ^2^-test with *Bonferroni’s* correction) in frequency of indels and SVs between large and small pigs, to understand their effects on body size. We found significant (*P* < 0.05) between-size-group differences for 10 indels and 20 SVs, located within 7 and 10 functional genes, respectively (Supplementary Tables S16 and S17). For all the seven small pigs, we identified a 4 bp insertion in the third exon of *COL1A1*. COL1A1 is an α1(I) protein chain of type I collagen and a major structural component of bone. Nonfunctional COL1A1 markedly reduces skeletal mineral density and body height^37,38^. We also found a 430 bp deletion in the third intron of the gene encoding propionyl CoA caboxylase α subunit (PCCA). A genetic defect in *PCCA* causes propionic acidemia, a condition that can lead to bone disease and growth failure^39^.

## Materials and Methods

### Samples

All animals used in this study were reared and euthanized with the approval of the College of Animal Science, South China Agricultural University. All experiments were performed in accordance with ‘*The Instructive Notions with Respect to Caring for Laboratory Animals*’, issued by the Ministry of Science and Technology of the People’s Republic of China. To clarify the genetic structure of Chinese pigs across different geographical locations, we selected individuals that represent all six Chinese indigenous types^4^: *South Chinese* (n = 10), *North China* (n = 2), *Lower Yangtze River Basin* (n = 2), *Central China* (n = 3), *Southwest* (n = 1), *Plateau* (n = 2). The proportion of representative breeds represented in our study from each type was shown in Supplementary Table S1. We also included samples from southern and northern Chinese wild pigs (n = 4), as well as European wild and commercial pigs (n = 7) (Table 1 and Supplementary Fig. S1). Altogether, data from 31 individual animals were used in this study: (i) 24 sampled from 22 breeds, which were handled by the South China Agricultural University, Guangzhou, People’s Republic of China (Table 1 and Supplementary Fig. S1) and (ii) seven (one pig per breed) downloaded from the Wageningen University Porcine Re-sequencing Phase 1 Project (http://www.ebi.ac.uk/ena/data/view/ERP001813)^12,17^ with the highest sequencing depths to supplement the breeds sampled here (Table 1). Seven small pigs and seven large pigs were used to detect candidate genes for body size (Supplementary Table S14). Body size data were obtained for 14 pigs, 11 from the book *Animal genetic resources in China: pigs*^21^, and three were measured according to the technical specifications for the registration of breeding pigs (NY/T 820-2004, 2004). A completed ARRIVE guidelines checklist is included in Table 1.

### DNA isolation and genome sequencing

Genomic DNA was extracted from ear tissue of live collection using a phenol-chloroform-based method. For each sample, 1–15 µg of DNA was sheared into 200–800 bp fragments using the Covaris system (Life Technologies). Fragments were then treated according to the Illumina DNA-sample-preparation protocol. For library construction, fragments were end-repaired, A-tailed, ligated to paired-end adaptors, and PCR-amplified with 500 bp inserts. Sequencing was performed to generate 100 bp paired-end reads on the HiSeq 2000 platform (Illumina), following the manufacturer’s protocol.

### Sequence alignment and genotype calling

Filtered reads were aligned to the Wuzhishan pig draft genome assembly (minipig_v1.0)^40^ using the Burrows-Wheeler Aligner^41^. This genome was selected as the reference^7,40^ after considering the geographical distance and genetic divergence among the 31 breeds (Table 1 and Supplementary Fig. S1 and Table S1).

Aligned bam files were sorted and indexed in Picard-tools version 1.117. Two GATK (Genome Analysis Toolkit version 2.4–9^42^ modules, RealignerTargetCreator and IndelRealigner), were used to realign the SNPs around indels in bam results. To obtain high-quality variants, additional GATK modules HaplotypeCaller and SAMtools^43^ were used to call variants for each sample. Only concordance variants were selected, and SNPs were filtered with the parameter “QD < 2.0 | | FS > 30.0 | | MQ < 40.0 | | DP < 6 | | DP > XXX | | ReadPosRankSum < -8.0 | | BaseQRankSum < -8,” while indels were filtered with “QD < 2.0 | | FS > 30.0 | | ReadPosRankSum < -8.0.” These variants were used to perform base quality score recalibration (BQSR), and resultant reads were applied calling population variants, done with the GATK HaplotypeCaller module using the parameter “–genotyping_mode DISCOVERY -stand_emit_conf 10 -stand_call_conf 30.”

To detect structural variants, we followed an existing method^44^, with some modifications. Reads were assembled into contigs and scaffolds using default parameters in SOAPdenovo. The assembled scaffold was mapped to the reference genome in BLAT^45^, with the –fastmap option.

Criteria for determining the most well-aligned scaffold included coverage length in a given region and high contig support. Selected scaffolds and reference-genome regions with the highest alignment were extracted and aligned to each other in LASTZ (http://www.bx.psu.edu/miller_lab/). Unmapped scaffolds were further aligned against the reference genome using BLASTn. Structural variants were extracted based on all aligned regions.

### Phylogenetic and population genetic analyses

Genetic structure was inferred from high-density SNP data in FRAPPE^46^, a program that applies maximum likelihood and expectation-maximization to estimate individual ancestry and admixture proportions. To explore individual convergence, we predefined the number of genetic clusters from K = 2 to K = 5. The maximum iteration of the expectation-maximization algorithm was set to 10,000.

A phylogenetic tree was generated from population-level SNPs in TreeBeST (http://treesoft.sourceforge.net/treebest.shtml), under the p-distances model. Population-level SNPs were then subjected to PCA in EIGENSOFT^47^, and eigenvectors were obtained using the R (https://www.r-project.org/) function eigen.

To evaluate LD decay, Haploview^48^ was used to calculate the squared correlation (*r*^2^) between any two loci. Average *r*^2^ was calculated for pairwise markers in a 5 Kb window and averaged across the whole genome. LD blocks were defined by the confidence interval method of Gabriel *et al*.^49^ and implemented in the Haploview 4.2 software (https://www.broadinstitute.org/haploview/haploview). Haplotype phase are inferred using a standard EM algorithm from the Haploview 4.2 software. The software script is as follows: “ava -jar Haploview.4.2.jar -n -pedfile X_112.ped -info X_112.info -maxdistance 500 -minMAF 0.0 -hwcutoff 0.001 -log X_112.log -blockoutput GAB -memory 19240 -pairwiseTagging -hwcutoff 0.00000”.

### Gene and QTL annotation

Pathway analyses of candidate genes were performed using KEGG (https://www.genome.jp/kegg/pathway.html). KEGG analysis is mainly performed by the following three steps: 1) Extract the nucleoside and protein sequences of the target gene, 2) Align the protein sequences to the KEGG animal database with the alignment software BLAST3, 3) Classify each gene according to the annotation information. Additionally, identified QTLs were functionally characterized using Pig QTLdb (https://www.animalgenome.org/cgibin/QTLdb/SS/index, Release 23, Apr 21, 2014), specifically with coordinate conversion of the Wuzhishan genome to the European-Duroc reference genome (Sscrofa10.2). Indels were matched to the InterPro database using EBI InterProScan (https://www.ebi.ac.uk/interpro/search/sequence-search).

### Introgression analysis

Methods described in a published study^50^ were used. We applied a likelihood ratio test to study the ancestral contribution of groups to the genome of each individual pig. All putative introgressions between group pairs (North China, South China, and Europe) were examined. For every 100 Kb window containing at least 10 SNPs and when at least three comparisons were possible per group, we calculated the ratio of the average sharing per pig with its own and another group. Regions with an average sharing ratio of <0.8 were defined as introgressions. Shared introgression frequency was plotted and tabulated. Introgression length and number per pig were also tabulated. Regions of extensive haplotype sharing (≥90% shared SNPs) were considered introgressed regions for each group pair.

## Supplementary information

Supplementary information.

## Data Availability

The datasets generated and analysed during the current study are available from the corresponding author on reasonable request.
