# Supplementary material for: Whole genome sequence analysis reveals genetic structure and X-chromosome haplotype structure in indigenous Chinese pigs
Source: Sci Rep. 2020 Jun 10;10:9433. doi: 10.1038/s41598-020-66061-2 (PMC7286894; doi:10.1038/s41598-020-66061-2)
Supplement: Supplementary file 1 — Supplementary information. [file 41598_2020_66061_MOESM1_ESM.pdf]

Whole genome sequence analysis reveals genetic structure and X-chromosome haplotype structure in indigenous Chinese pigs

Xiong Tong,[†,1,3] Lianjie Hou,[†,1] Weiming He,[2] Chugang Mei,[4] Bo Huang,[1] Chi Zhang,[2] Chingyuan Hu,[5] and Chong Wang[*,1]

Supplemental Figures

Figure S1 Geographic distribution of 31 pigs in this study

Figure S2 Distribution of effective sequencing depth in all samples

Figure S3 Quality score of bases from all the reads

Figure S4 Structural annotation of SNPs

Figure S5 Structural annotation of Indels

Figure S6 The phylogenetic tree with boostrap values

Figure S7 Introgressed regions detected in pig chromosome 5, 14, 17 and 18

Figure S8 The diversity (θπ/Kb) and divergence index (F_{ST}) across the populations

Figure S9 LD decay patterns of different populations

Supplemental Tables

Table S1 Proportion of representative breeds from each type

Table S2 Summary of the mapping reads

Table S3 Detection and statistics of SNPs in all samples

Table S4 Detection and statistics of Indels in all samples

Table S5 Detection and statistics of SVs in all samples

Table S6 TW (Tracy-Widom) statistics for the first eight eigenvalues

Table S7 Statistics of θπ and θw in different populations

Table S8 Summary of annotated genes in the 40.09 Mb LD block

Table S9 Functional gene categories enriched for genes on the LD block region

Table S10 Overlapping QTLs with the LD block region on chromosome X

Table S11 Indels exhibited significant difference between domestic populations of south china and north china

Table S12 Top 100 SVs with different distribution (χ^2 with *FDR* correction, $P < 0.01$) between CnNorth and CnSouth

Table S13 SVs exhibited significant difference between domestic populations of south china and north china

Table S14 Adult body sizes from small and large pigs in this study

Table S15 Candidate genes detected in accordance with >80% in one group and <20% in the other

Table S16 Indels exhibited significant difference between pig populations of big size and small size

Table S17 SVs exhibited significant difference between pig populations of big size and small size

SUPPLEMENTARY FIGURES

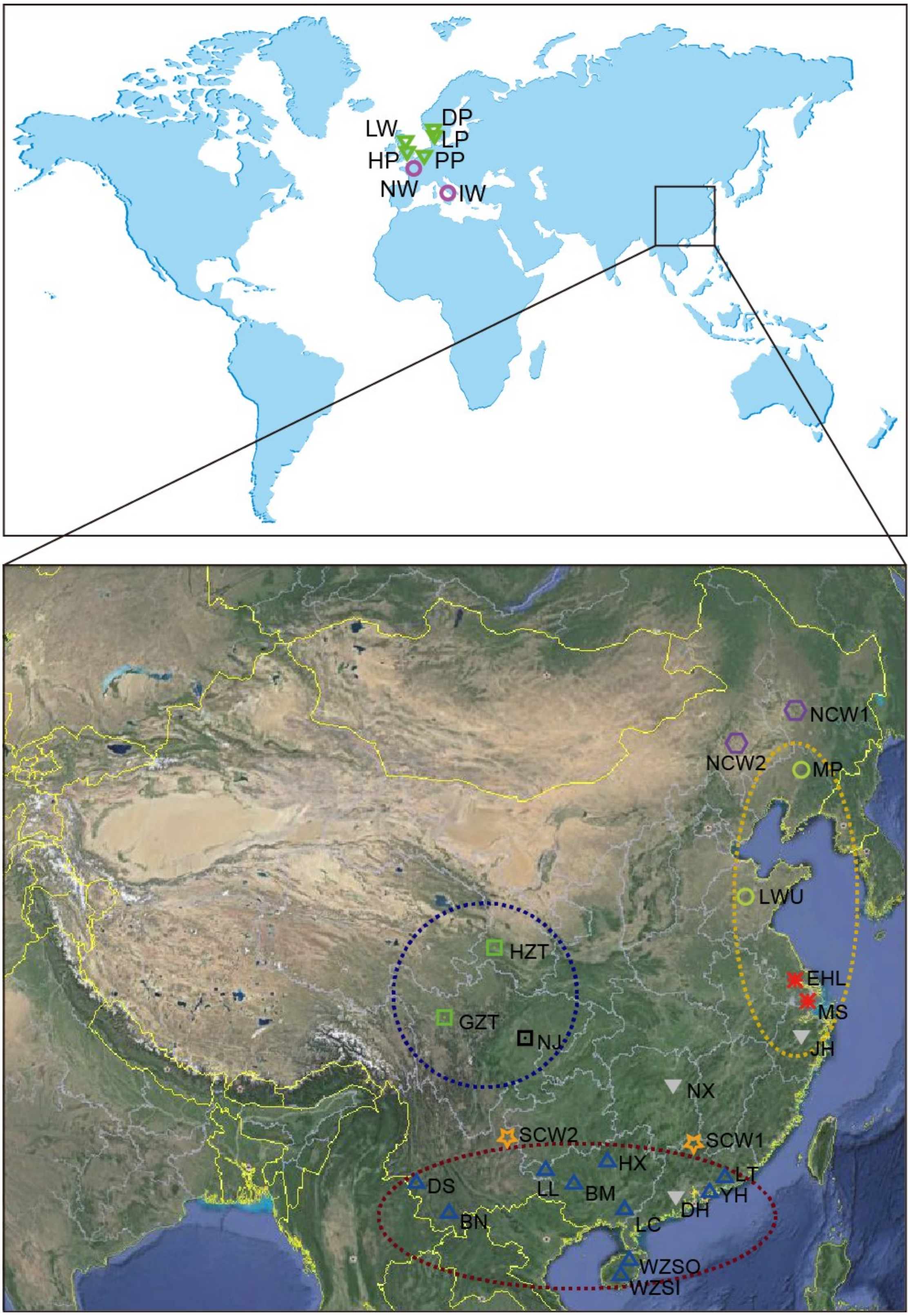

Figure S1. Geographic distributions of 31 pigs in this study. These pigs represent all of the six types of Chinese indigenous breeds (blue triangle, *South Chinese Type*: HX, YH, BM, LC, WZSO, WZSI, LT, LL, DS and BN, n=9; yellowish circle, *North China Type*: MP and LWU, n=2; red

cross-over line, *Lower Yangtze River Basin Type*: EHL and MS, n=2; white inverted triangle, *Central China Type*: DH, JH and NX, n=3; black square, *Southwest Type*: NJ, n=1; green square, *Plateau Type* : GZT and HZT, n=2) and South and North China wild boars (yellow pentagon, South China wild boars: SCW1 and SCW2, n=2; purple hexagon, North China wild boars: NCW1 and NCW2, n=2), and major European boars (purple circle, NW and IW, n=2) and commercial breeds (green inverted triangle, LW, HP, DP, LP and PP, n=5). Geographical distributions of the three subgroups in Chinese domestic pigs are represented by different dotted circles (red dotted circle, CnSouth; blue dotted circle, Subgroup 2; yellow dotted circle, Subgroup 1). This map was generated by Google Earth (Image @2018 DigitalGlobe, https://www.google.com/permissions/geoguidelines/attr-guide.html) and edited by adobe illustrator CS6 software (https://www.adobe.com/cn/products/illustrator/free-trial-download.html).

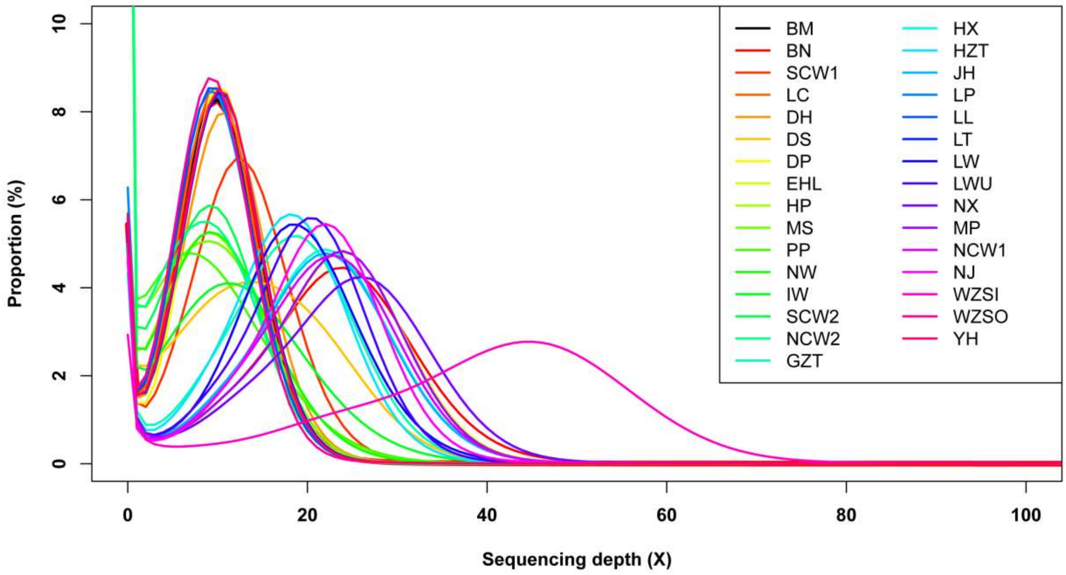

Figure S2. Distribution of effective sequencing depth in all samples. Whole-genome resequencing information of 24 sampled pigs from 22 breeds of China was with an average effective sequencing depth of 17.54×. The average sequencing depth of seven samples (one pig per breed) from the Wageningen University Porcine re-sequencing Phase 1 Project) was almost 14.5×, which is a bit lower than the samples be sequenced in this study.

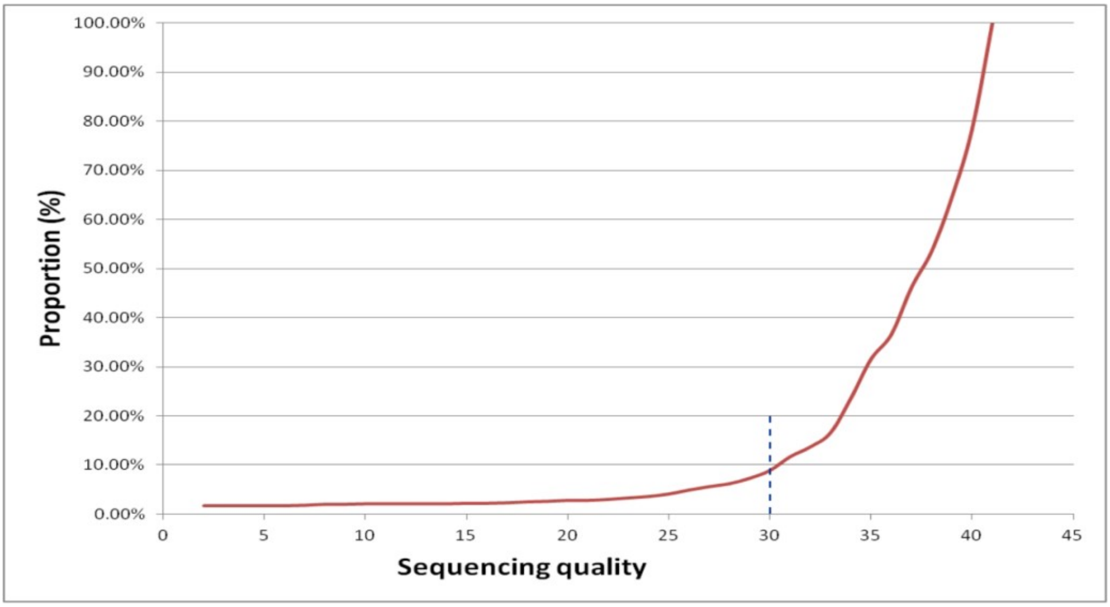

Figure S3. Quality score of bases from all the reads. The quality of more than 90% of the reads was above Q30.

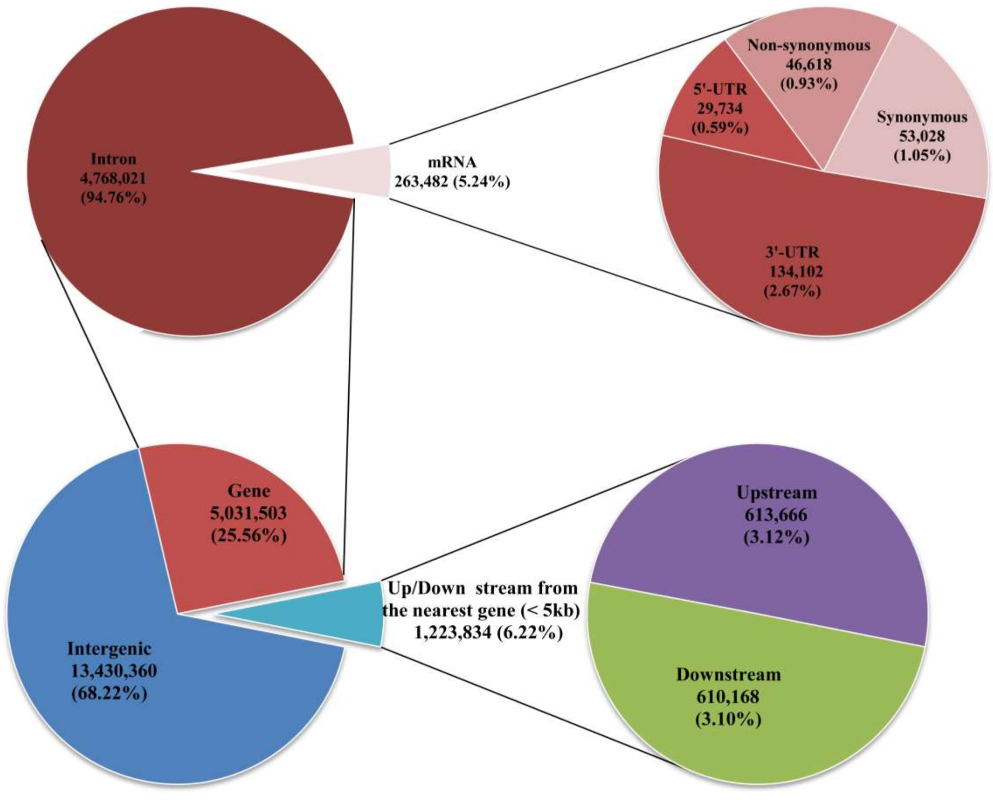

Figure S4. Structural annotation of SNPs.

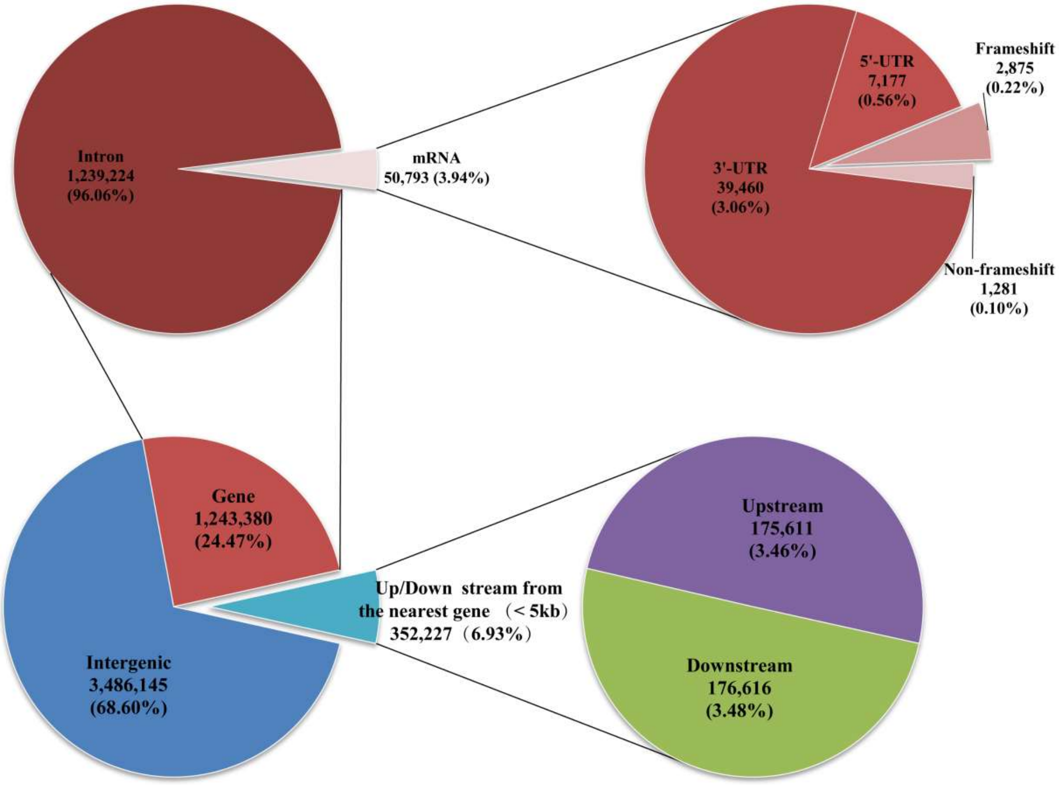

Figure S5. Structural annotation of Indels.

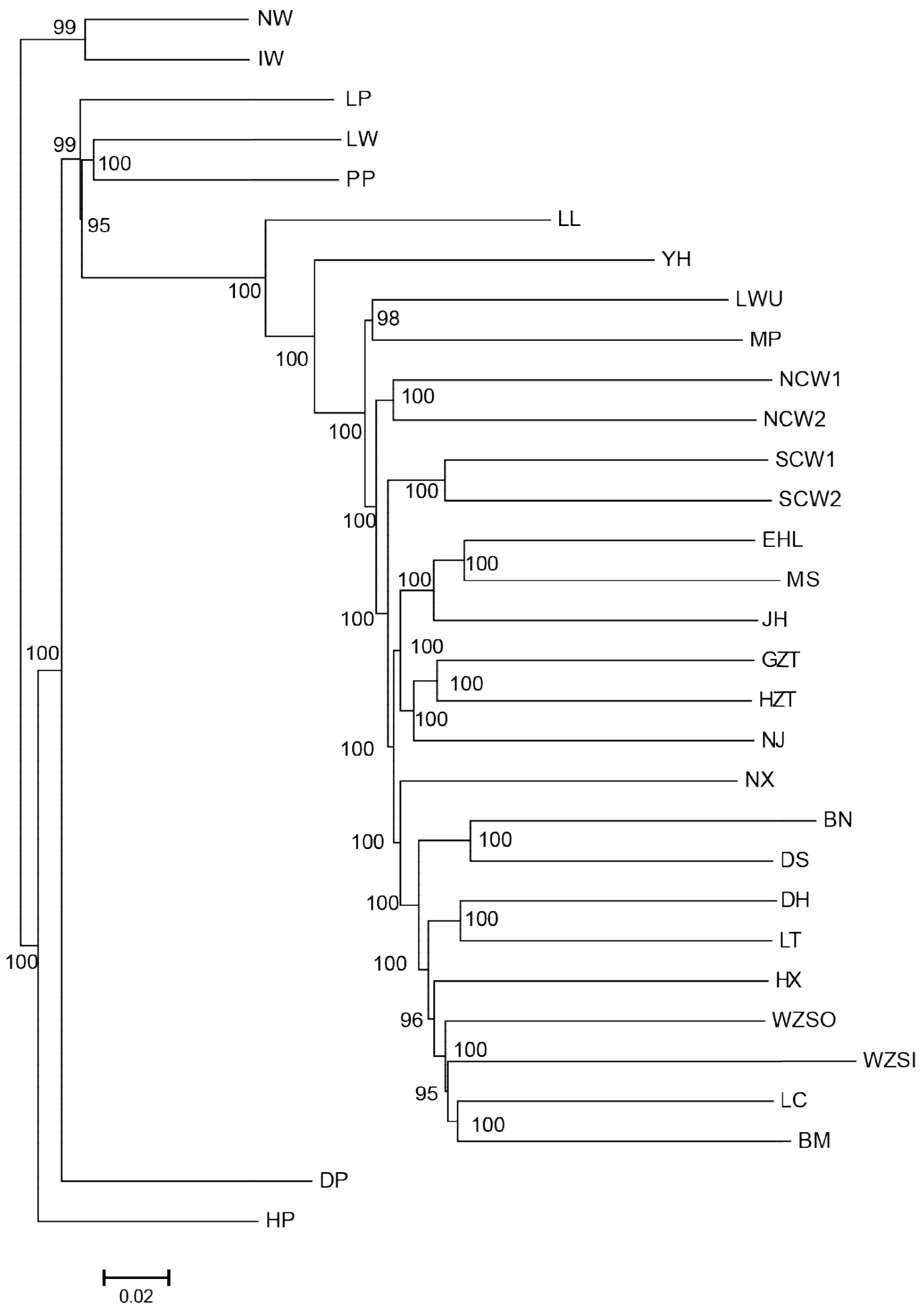

Figure S6. The phylogenetic tree with boostrap values

a.

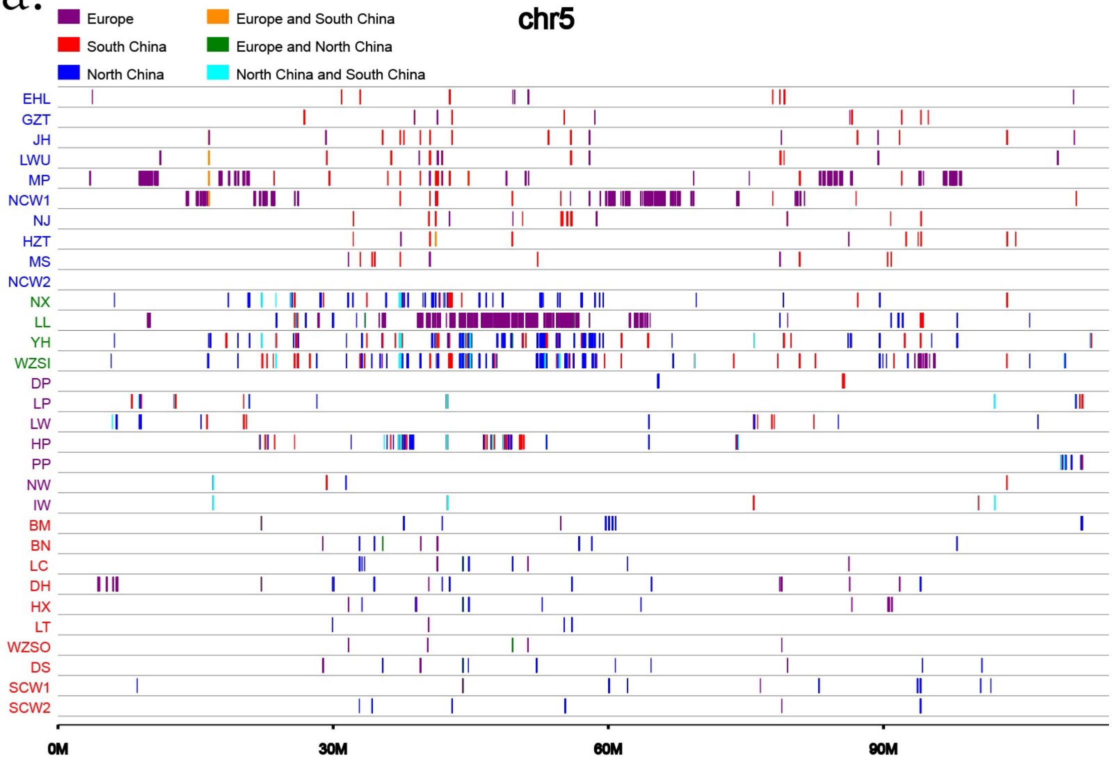

chr5
Europe
South China
North China
Europe and South China
Europe and North China
North China and South China
EHL
GZT
JH
LWU
MP
NCW1
NJ
HZT
MS
NCW2
NX
LL
YH
WZSI
DP
LP
LW
HP
PP
NW
IW
BM
BN
LC
DH
HX
LT
WZSO
DS
SCW1
SCW2
0M
30M
60M
90M

b.

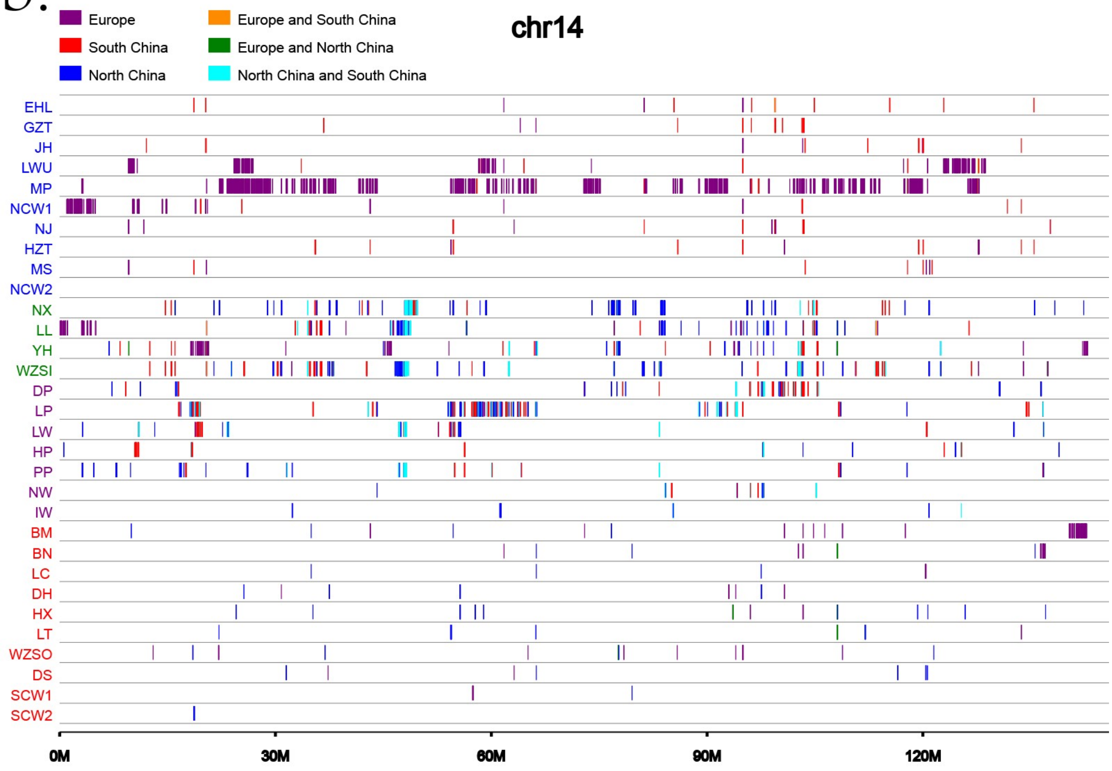

chr14
Europe
South China
North China
Europe and South China
Europe and North China
North China and South China
EHL
GZT
JH
LWU
MP
NCW1
NJ
HZT
MS
NCW2
NX
LL
YH
WZSI
DP
LP
LW
HP
PP
NW
IW
BM
BN
LC
DH
HX
LT
WZSO
DS
SCW1
SCW2
0M
30M
60M
90M
120M

c.

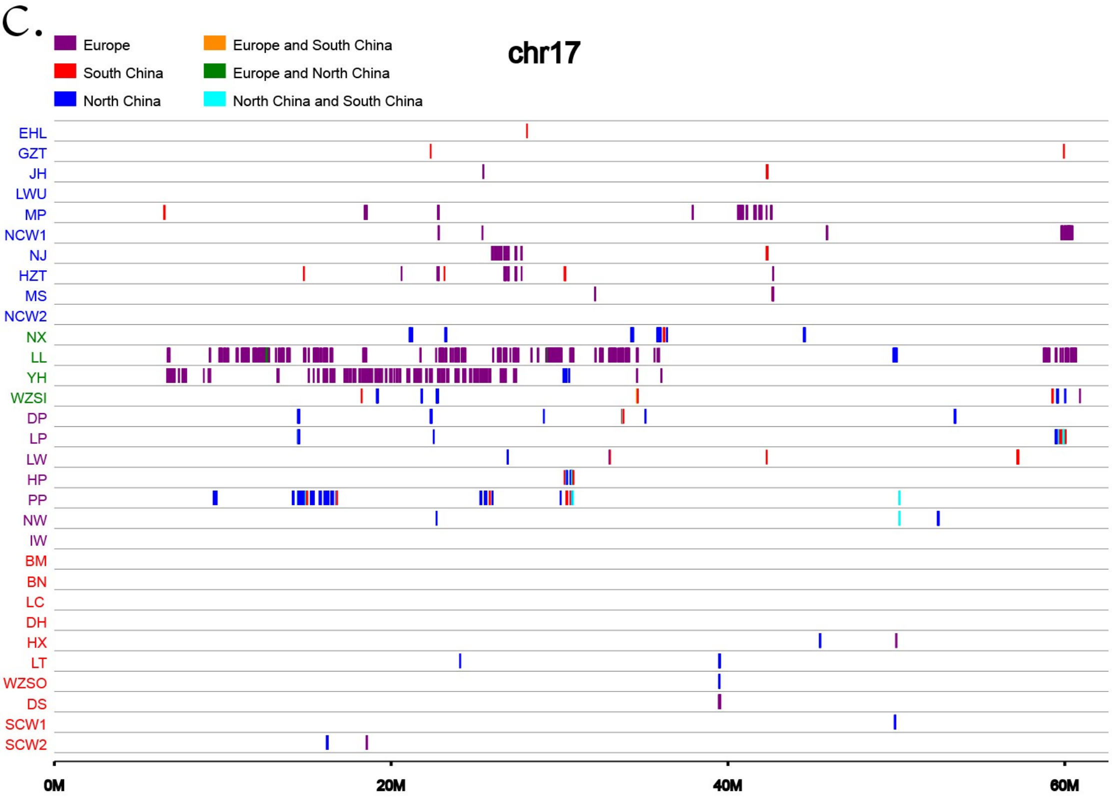

d.

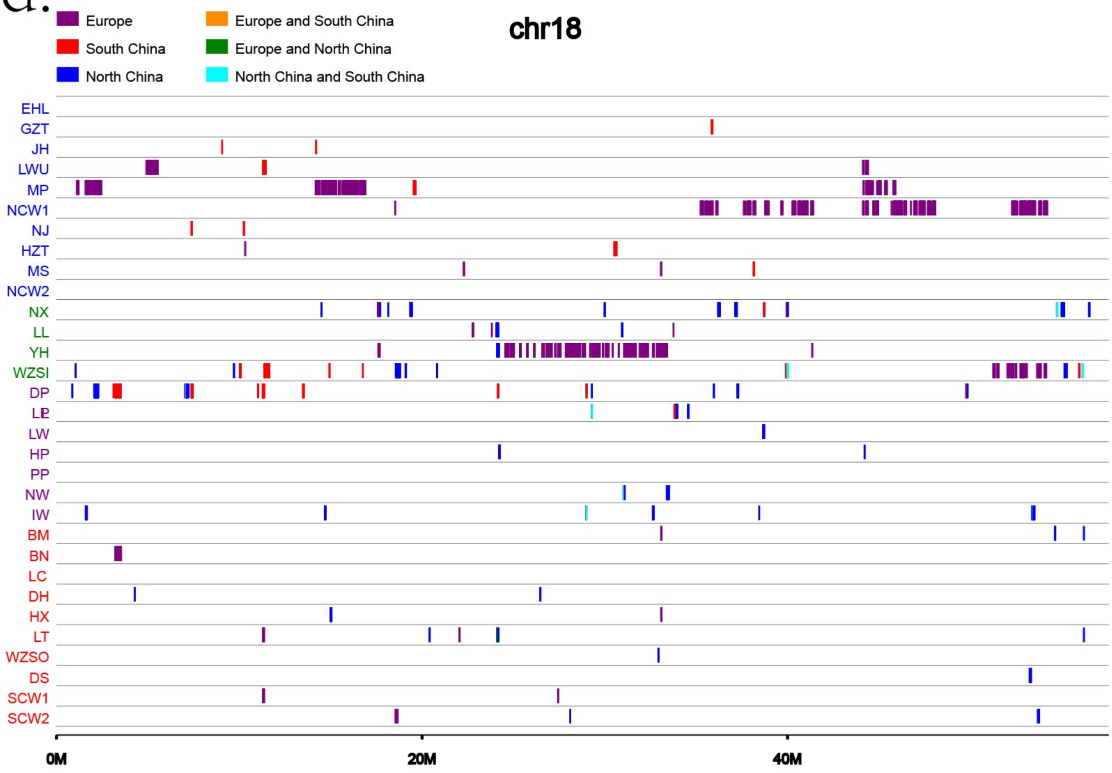

Figure S7. Introgressed regions detected in pig chromosome 5 (a), 14 (b), 17 (c) and 18 (d). The origin of an introgression is represented by the color of the specific pig population (red from South China, blue from North China, purple from Europe, blue|red from North China and South

China, purple|red from Europe and South China, and bule|purple from North China and Europe). Five Chinese breeds (LWU, MP, LL, YH and NCW1) harbor multiple and densely covered regions on chromosomes 5, 14, 17, and 18 that were introgressed from the European population.

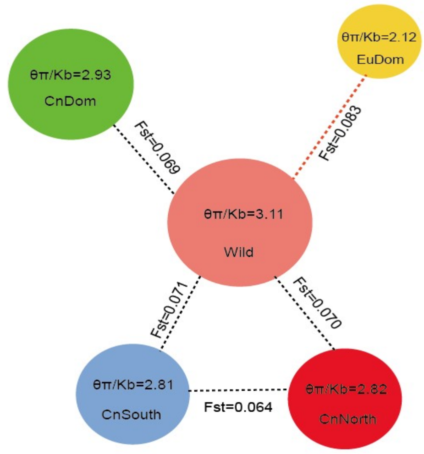

Figure S8. The diversity (θπ/Kb) and divergence index (F_{ST}) across the populations. Different colored balls represent different populations. The radius of pie represents the genetic diversity of different populations. Dashed line length represents F_{ST} value between two populations.

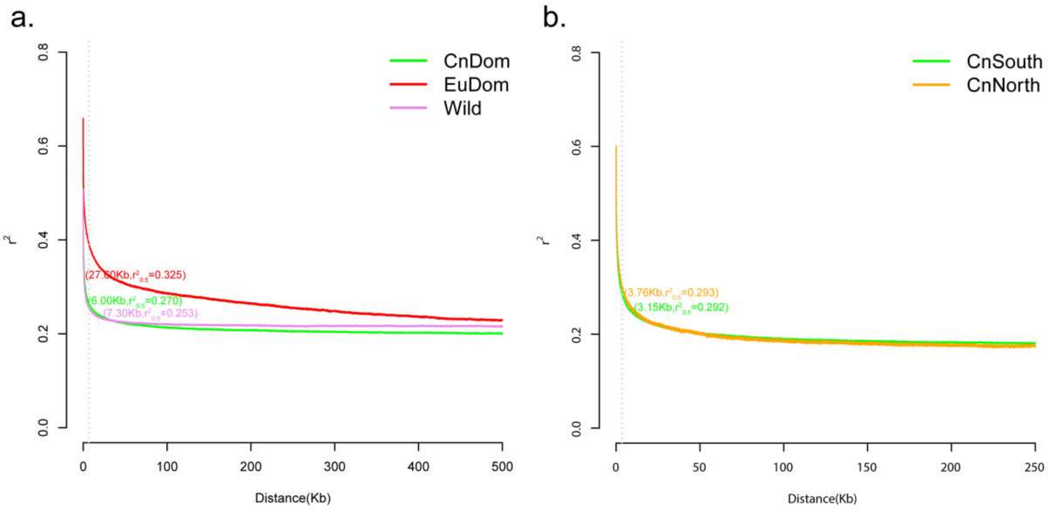

Figure S9. LD decay patterns of different populations. (a) LD decay patterns of EuDom (European domestic pigs), CnDom (Chinese domestic pigs), and Wild (Wild boars). (b) LD decay patterns of CnSouth (subpopulation of South China in the Chinese domestic pigs) and CnNorth (subpopulation of North China in the Chinese domestic pigs). Each curve with a label: (LD distance, $r^2_{0.5}$ value). The LD analysis revealed that the Europe pigs were with longer LD block size than the wild pigs and Chinese pigs, and the two subpopulations of china (CnNorth and CnSouth) exhibit similar levels of nucleotide diversity.

SUPPLEMENTARY TABLES

Table S1. Proportion of representative breeds from each type.

Classification Type	Total number of Recognized Breeds* (n)	Breeds in our study# (n)	Proportion (%)
South Chinese	9	10 (including WZSI)	100
Central China	19	3	15.79
North China	5	2	40
Lower Yangtze River Basin	7	2	28.57
Southwest	7	1	14.29
Plateau	1	2 (including two regions)	100
Total	48	20	41.67

Notes: Chinese indigenous pigs (except for wild boars) are historically classified into 48 breeds and split into six types (*South Chinese*, *North China*, *Lower Yangtze River Basin*, *Central China*, *Southwest*, and *Plateau*), based on geographic distribution, historical origin, and morphological characteristics. In this study, representative breeds of each type were selected based on the phylogenetic tree constructed by microsatellite markers and target traits.

(1) * Number of representative breeds from each type in the book *Pig breeds in China*[4].

(2) # Number of representative breeds from each type in this study.

Table S2. Summary of the mapping reads.

Classification Types	Sample	Raw Reads (M)	Mapping rate (%)			Depth（×）	Genome Coverage (%)
			Total reads	PE reads*	Uniquely mapped reads		
South Chinese (n=10)	BM	323	95.66	90.02	87.21	10.76	94.57
	BN	710	95.42	91.82	87.04	23.58	95.26
	DS	475	95.64	90.10	86.38	15.82	93.89
	HX	635	95.33	87.85	88.41	22.12	95.67
	LC	312	96.40	91.94	87.69	10.45	94.30
	LL	315	95.24	90.17	86.70	10.42	94.42
	LT	324	96.06	90.26	87.67	10.82	94.34
	WZSI	1,145	98.02	92.79	91.39	41.21	97.07
	WZSO	299	95.83	91.47	87.60	9.98	94.38
	YH	323	95.66	91.31	87.32	10.75	94.35
Central China (n=3)	DH	344	95.04	90.98	86.92	11.37	94.50
	JH	636	97.34	92.79	89.20	21.55	94.55
	NX	715	97.17	93.77	88.84	24.16	94.81
North China (n=2)	LWU	579	96.89	93.40	89.63	19.52	94.65
	MP	654	97.25	94.15	90.05	22.12	94.54
Lower Yangtze River Basin (n=2)	EHL	676	97.04	92.24	89.15	22.83	95.01
	MS	205	99.59	98.81	96.09	7.41	77.55
Southwest (n=1)	NJ	626	97.21	92.30	89.63	21.18	95.27
Plateau (n=2)	GZT	554	94.18	93.02	89.17	18.14	94.53
	HZT	547	95.98	91.62	87.00	18.25	95.05
European domestic breeds (n=5)	DP	326	95.33	91.71	87.23	10.82	93.87
	HP	235	99.28	99.07	95.26	8.42	78.16
	LP	309	94.75	91.02	86.81	10.18	93.73
	LW	582	95.42	91.53	87.55	19.32	94.64
	PP	223	99.29	98.94	95.13	7.89	76.77
Wild boar (n=6)	IW	291	99.32	99.11	94.76	10.50	78.04
	NCW1	666	96.31	91.29	88.15	22.33	95.39
	NCW2	209	99.48	98.92	95.12	7.27	77.39
	NW	241	99.22	98.89	94.99	8.50	78.36
	SCW1	404	94.97	90.02	85.86	13.35	94.87
	SCW2	209	99.47	99.01	95.73	7.42	77.64

Abbreviations: PE reads, paired-end reads.

Table S3. Detection and statistics of SNPs in all samples.

Classification Types	Sample	SNP					
		Homo	Homo (%)	Hete	Hete (%)	Total	Homo/Hete
South Chinese (n=10)	BM	3,441,165	0.151	2,885,182	0.126	6,326,347	1.193
	BN	5,621,174	0.235	3,695,833	0.155	9,317,007	1.521
	DS	3,492,528	0.156	5,304,906	0.237	8,797,434	0.658
	HX	919,624	0.038	1,070,856	0.045	1,990,480	0.859
	LC	3,161,759	0.139	3,769,787	0.165	6,931,546	0.839
	LL	2,880,153	0.127	5,478,777	0.241	8,358,930	0.526
	LT	3,330,506	0.145	3,923,841	0.171	7,254,347	0.849
	WZSI	95,979	0.004	2,781,302	0.117	2,877,281	0.035
	WZSO	2,366,368	0.104	4,208,300	0.186	6,574,668	0.562
	YH	3,356,234	0.146	4,770,083	0.208	8,126,317	0.704
Central China (n=3)	DH	3,463,050	0.150	4,267,714	0.185	7,730,764	0.811
	JH	5,577,519	0.235	5,091,843	0.215	10,669,362	1.095
	NX	4,491,346	0.190	7,044,898	0.298	11,536,244	0.638
North China (n=2)	LWU	5,635,122	0.237	5,165,887	0.218	10,801,009	1.091
	MP	5,681,589	0.240	5,763,631	0.243	11,445,220	0.986
Lower Yangtze River Basin (n=2)	EHL	5,305,469	0.223	5,788,000	0.244	11,093,469	0.917
	MS	2,185,820	0.127	2,074,846	0.121	4,260,666	1.053
Southwest (n=1)	NJ	5,379,738	0.224	5,967,273	0.249	11,347,011	0.902
Plateau (n=2)	GZT	5,040,423	0.214	5,541,325	0.235	10,581,748	0.910
	HZT	4,364,314	0.184	5,886,206	0.248	10,250,520	0.741
European domestic breeds (n=5)	DP	5,082,935	0.221	2,383,559	0.104	7,466,494	2.132
	HP	418,480	0.023	179,973	0.010	598,453	2.325
	LP	4,642,705	0.206	2,827,897	0.125	7,470,602	1.642
	LW	6,700,029	0.282	3,657,514	0.154	10,357,543	1.832
	PP	155,659	0.009	95,668	0.006	251,327	1.627
Wild boar (n=6)	IW	5,579,689	0.303	1,250,996	0.068	6,830,685	4.460
	NCW1	5,690,633	0.238	5,998,401	0.251	11,689,034	0.949
	NCW2	2,432,758	0.142	2,736,961	0.160	5,169,719	0.889
	NW	4,947,588	0.273	1,300,658	0.072	6,248,246	3.804
	SCW1	3,709,425	0.159	5,370,929	0.230	9,080,354	0.691
	SCW2	2,231,048	0.127	3,668,166	0.209	5,899,214	0.608

Abbreviations: Homo, homozygous SNPs; Hete, heterozygous SNPs; Homo/Hete, ratio of homozygous/heterozygous SNPs.

Table S4. Detection and statistics of Indels in all samples.

Classification Types	Sample	Indel					
		Homo	Homo (%)	Hete	Hete (%)	Total	Homo/Hete
South Chinese (n=10)	BM	547,413	0.024	560,789	0.025	1,108,202	0.976
	BN	748,216	0.031	1,153,685	0.048	1,901,901	0.649
	DS	476,326	0.021	994,477	0.044	1,470,803	0.479
	HX	550,819	0.023	1,241,163	0.052	1,791,982	0.444
	LC	488,176	0.021	641,698	0.028	1,129,874	0.761
	LL	442,407	0.019	780,586	0.034	1,222,993	0.567
	LT	510,463	0.022	673,535	0.029	1,183,998	0.758
	WZSI	32,396	0.001	392,659	0.017	425,055	0.083
	WZSO	409,454	0.018	657,718	0.029	1,067,172	0.623
	YH	515,698	0.022	758,203	0.033	1,273,901	0.680
Central China (n=3)	DH	507,924	0.022	725,621	0.032	1,233,545	0.700
	JH	707,246	0.030	1,178,794	0.050	1,886,040	0.600
	NX	558,332	0.024	1,444,970	0.061	2,003,302	0.386
North China (n=2)	LWU	722,660	0.030	1,166,429	0.049	1,889,089	0.620
	MP	710,940	0.030	1,308,689	0.055	2,019,629	0.543
Lower Yangtze River Basin (n=2)	EHL	669,128	0.028	1,274,940	0.054	1,944,068	0.525
	MS	397,286	0.023	370,565	0.022	767,851	1.072
Southwest (n=1)	NJ	697,145	0.029	1,320,296	0.055	2,017,441	0.528
Plateau (n=2)	GZT	644,542	0.027	1,129,065	0.048	1,773,607	0.571
	HZT	598,048	0.025	1,311,950	0.055	1,909,998	0.456
European domestic breeds (n=5)	DP	792,379	0.034	611,666	0.027	1,404,045	1.295
	HP	65,048	0.004	33,722	0.002	98,770	1.929
	LP	693,546	0.031	569,131	0.025	1,262,677	1.219
	LW	905,393	0.038	1,097,107	0.046	2,002,500	0.825
	PP	25,211	0.001	16,645	0.001	41,856	1.515
Wild boar (n=6)	IW	758,893	0.041	359,166	0.019	1,118,059	2.113
	NCW1	721,019	0.030	1,308,049	0.055	2,029,068	0.551
	NCW2	401,471	0.023	390,456	0.023	791,927	1.028
	NW	677,596	0.037	302,392	0.017	979,988	2.241
	SCW1	534,566	0.023	1,005,332	0.043	1,539,898	0.532
	SCW2	362,594	0.021	459,582	0.026	822,176	0.789

Abbreviations: Homo, homozygous Indels; Hete, heterozygous Indels; Homo/Hete, ratio of homozygous/heterozygous Indels.

Table S5. Detection and statistics of SVs in all samples.

Classification Types	Sample	DEL	INS	INV	ITX	CTX	Total
South Chinese (n=10)	BM	16,346	0	68	19	7,842	24,275
	BN	26,943	91	131	76	22,698	49,939
	DS	20,706	13	107	60	14,336	35,222
	HX	2,845	1	24	11	0	2,881
	LC	16,312	23	52	28	7,979	24,394
	LL	15,303	14	62	31	7,510	22,920
	LT	13,464	20	58	25	4,683	18,250
	WZSI	4,282	14	76	55	26,060	30,487
	WZSO	13,939	15	48	31	7,006	21,039
	YH	16,239	14	57	20	7,828	24,158
Central China (n=3)	DH	16,589	12	55	17	8,244	24,917
	JH	23,303	28	113	34	17,857	41,335
	NX	22,613	45	121	42	20,778	43,599
North China (n=2)	LWU	24,243	39	112	43	17,846	42,283
	MP	21,445	17	121	38	410	22,031
Lower Yangtze River Basin (n=2)	EHL	25,166	0	122	47	18,371	43,706
	MS	9,649	0	11	4	2,113	11,777
Southwest (n=1)	NJ	21,170	46	109	49	16,309	37,683
Plateau (n=2)	GZT	18,314	1	97	31	9,764	28,207
	HZT	22,338	17	91	38	15,563	38,047
European domestic breeds (n=5)	DP	18,904	6	74	25	6,995	26,004
	HP	2,369	0	16	4	1,564	3,953
	LP	17,889	0	67	23	6,260	24,239
	LW	25,172	18	118	53	15,410	40,771
	PP	3,164	0	16	9	1,895	5,084
Wild boar (n=6)	IW	3,475	0	28	6	2,716	6,225
	NCW1	20,282	54	133	50	17,240	37,759
	NCW2	7,186	0	14	5	1,783	8,988
	NW	4,001	0	21	8	1,948	5,978
	SCW1	20,506	0	62	31	10,432	31,031
	SCW2	4,791	0	13	8	1,570	6,382

Abbreviations: DEL, deletion; INS, insertion; INV, inversion; ITX, intra-chromosomal translocation; CTX, Inter-chromosomal translocation.

Table S6. TW (Tracy-Widom) statistics for the first eight eigenvalues.

Number	Eigenvalue	TW statistics	*P* value
1	14.167	6.766	2.58×10^{-7}
2	11.279	12.860	3.31×10^{-15}
3	4.782	9.124	8.50×10^{-10}
4	3.632	2.839	2.32×10^{-3}
5	3.423	2.756	2.71×10^{-3}
6	3.164	1.411	2.67×10^{-2}
7	2.911	-0.925	3.94×10^{-1}
8	2.857	-0.344	2.39×10^{-1}

Table S7. Statistics of θπ and θ_w in different populations.

Statistic	Wild	EuDom	CnDom	CnSouth	CnNorth
θ_π/Kb	3.11	2.12	2.93	2.81	2.82
θ_w/Kb	2.80	2.01	2.52	2.53	2.68

Note: Wild (Wild boars), CnDom (Chinese domestic pigs), EuDom (European domestic pigs), CnSouth (subpopulation of South China in the Chinese domestic pigs), and CnNorth (subpopulation of North China in the Chinese domestic pigs).

Table S8. Summary of annotated genes in the 40.09 Mb LD block.

Chr	Gene Name	Gene ID	QTL_Number	Contained SNPs	Exonic Function
X	AWAT1	WZSP003173	14		
X		ENSSSCT00000013538	14		
X	ARR3	WZSP003172	14		
X	PDZD11	WZSP003171	14		
X	KIF4A	WZSP003170	14		
X	GDPD2	WZSP003169	14		
X	DLG3	WZSP003168	14		
X		ENSSSCT00000034690	14		
X	BMP15	WZSP005447	10	with SNP	non-synonymous SNP
X	ERVK6	WZSP005448	10	with SNP	non-synonymous SNP
X		ENSSSCT00000013462	10		
X	GSPT2	WZSP015978	10		
X	MAGED1	WZSP015977	10	with SNP	
X	MAGED4	WZSP006461	10	with SNP	non-synonymous SNP
X	FAM156A	WZSP015990	10		
X	NA	WZSP015989	10		
X	GPR173	WZSP001342	10	with SNP	
X	TSPYL2	WZSP001341	10	with SNP	non-synonymous SNP
X	KDM5C	WZSP001340	10	with SNP	
X	IQSEC2	WZSP001339	10	with SNP	
X	SMC1A	WZSP000023	10	with SNP	
X	RIBC1	WZSP000022	10	with SNP	non-synonymous SNP
X	HSD17B10	WZSP000021	10	with SNP	non-synonymous SNP
X	HUWE1	WZSP000020	10	with SNP	non-synonymous SNP
X		ENSSSCT00000024962	10		
X	PHF8	WZSP001987	10	with SNP	non-synonymous SNP
X		ENSSSCT00000031381	10	with SNP	non-synonymous SNP
X	FAM120C	WZSP001986	10		
X	WNK3	WZSP001985	10	with SNP	non-synonymous SNP
X	TSR2	WZSP001984	10	with SNP	non-synonymous SNP
X	FGD1	WZSP001983	11	with SNP	non-synonymous SNP
X	GNL3L	WZSP001982	11	with SNP	non-synonymous SNP
X	ITIH5L	WZSP015988	11	with SNP	non-synonymous SNP
X	MAGED2	WZSP015987	11	with SNP	non-synonymous SNP
X	TRO	WZSP011139	11	with SNP	non-synonymous SNP
X	PFKFB1	WZSP011140	11	with SNP	
X	APEX2	WZSP011141	11	with SNP	non-synonymous SNP
X	ALAS2	WZSP011142	11	with SNP	non-synonymous SNP
X	PAGE2B	WZSP011143	11	with SNP	non-synonymous SNP
X	USP51	WZSP007653	11	with SNP	non-synonymous SNP
X	KLF8	WZSP016698	11		
X	SPIN2	WZSP002818	11		
X	GMCL1L	WZSP002827	11	with SNP	non-synonymous SNP
X	ZXDB	WZSP003459	11	with SNP	non-synonymous SNP
X	SPIN4	WZSP013117	11		
X	ARHGEF9	WZSP013116	11	with SNP	non-synonymous SNP
X	FAM123B	WZSP016710	11	with SNP	non-synonymous SNP
X	ASB12	WZSP016709	11		
X	MTMR8	WZSP016708	11	with SNP	
X		ENSSSCT00000013527	11		
X	ZC4H2	WZSP016707	11	with SNP	non-synonymous SNP
X	ZC3H12B	WZSP013815	11	with SNP	non-synonymous SNP
X	LAS1L	WZSP013814	11	with SNP	non-synonymous SNP
X	MSN	WZSP013813	14	with SNP	
X	VSIG4	WZSP003801	15	with SNP	non-synonymous SNP
X	HEPH	WZSP003802	15	with SNP	non-synonymous SNP
X		ENSSSCT00000028435	16	with SNP	non-synonymous SNP
X	EDA2R	WZSP003803	16		
X	NA	WZSP018773	18	with SNP	non-synonymous SNP
X		ENSSSCT00000013528	19	with SNP	
X	HNRNPA3	WZSP004763	18	with SNP	non-synonymous SNP
X	OPHN1	WZSP004764	18	with SNP	non-synonymous SNP
X		ENSSSCT00000033803	18	with SNP	
X		ENSSSCT00000014024	16		
X	YIPF6	WZSP004765	16		
X	STARD8	WZSP004766	15	with SNP	non-synonymous SNP
X	EFNB1	WZSP004767	15	with SNP	
X	PJA1	WZSP004768	15	with SNP	non-synonymous SNP

X		ENSSSCT00000013544	15		
X	EDA	WZSP004769	14	with SNP	
X	AWAT2	WZSP004770	14	with SNP	non-synonymous SNP
X	OTUD6A	WZSP004771	14		
X	IGBP1	WZSP004772	14	with SNP	non-synonymous SNP
X	DGAT2L6	WZSP004773	14	with SNP	
X		ENSSSCT00000027081	14	with SNP	non-synonymous SNP
X	TEX11	WZSP003281	14		
X	SLC7A3	WZSP003280	14	with SNP	non-synonymous SNP
X	UBE2E1	WZSP003279	14	with SNP	non-synonymous SNP
X	SNX12	WZSP003277	14		
X	NA	WZSP003278	14		
X	FOXO4	WZSP003276	14	with SNP	non-synonymous SNP
X	NA	WZSP003275	14		
X	IL2RG	WZSP003274	14	with SNP	
X	MED12	WZSP003273	14	with SNP	non-synonymous SNP
X	NLGN3	WZSP003272	14	with SNP	non-synonymous SNP
X	GJB1	WZSP003271	14	with SNP	
X	ZMYM3	WZSP003270	14		
X	POL	WZSP003269	14	with SNP	non-synonymous SNP
X	NONO	WZSP003268	14	with SNP	
X	ITGB1BP2	WZSP003267	14		
X	RHOG	WZSP003266	14	with SNP	
X	TAF1	WZSP003265	14	with SNP	non-synonymous SNP
X	OGT	WZSP003264	14	with SNP	
X	ACRC	WZSP003263	14	with SNP	non-synonymous SNP
X	CXCR3	WZSP003262	14	with SNP	
X	NA	WZSP005996	14	with SNP	non-synonymous SNP
X	NA	WZSP005995	14	with SNP	non-synonymous SNP
X		ENSSSCT00000022492	14		
X	NHSL2	WZSP005994	14	with SNP	non-synonymous SNP
X	PIN4	WZSP005993	14	with SNP	non-synonymous SNP
X		ENSSSCT00000023457	14	with SNP	non-synonymous SNP
X		ENSSSCT00000013565	14	with SNP	non-synonymous SNP
X	RPS4	WZSP005992	14	with SNP	
X	CITED1	WZSP005991	14		
X	NA	WZSP005990	14	with SNP	non-synonymous SNP
X	HDAC8	WZSP005989	14		
X	PHKA1	WZSP005988	14	with SNP	
X	NAP1L2	WZSP005304	14	with SNP	non-synonymous SNP
X	NAP1L2	WZSP005303	14	with SNP	non-synonymous SNP
X	ZNF345	WZSP005302	14		
X	CDX4	WZSP005301	14	with SNP	
X	CHIC1	WZSP005300	14	with SNP	
X	RLIM	WZSP019388	14		
X	KIAA2022	WZSP019389	14		
X	ABCB7	WZSP019390	14	with SNP	non-synonymous SNP
X	UPRT	WZSP019391	14	with SNP	non-synonymous SNP
X	ZDHHC15	WZSP019392	14	with SNP	non-synonymous SNP
X		ENSSSCT00000013591	14	with SNP	non-synonymous SNP
X	CXORF26	WZSP015072	14	with SNP	non-synonymous SNP
X	MAGEE2	WZSP015073	14		
X	POL	WZSP003974	14		
X	GAG	WZSP003975	14		
X	FGF16	WZSP003976	14		
X	ATRX	WZSP003977	14	with SNP	non-synonymous SNP
X	MAGT1	WZSP003978	14	with SNP	
X	COX7B	WZSP003979	14	with SNP	non-synonymous SNP
X	ATP7A	WZSP003980	14	with SNP	non-synonymous SNP
X	CYCS	WZSP003981	14	with SNP	non-synonymous SNP
X	TAF9B	WZSP015214	14		
X	PGK1	WZSP015213	14	with SNP	
X	CYSLTR1	WZSP010798	14	with SNP	non-synonymous SNP
X	ZCCHC5	WZSP016717	14	with SNP	non-synonymous SNP
X	BRWD3	WZSP010339	14	with SNP	non-synonymous SNP
X	NSBP1	WZSP010338	14	with SNP	non-synonymous SNP
X	SH3BGRL	WZSP010337	14	with SNP	non-synonymous SNP
X	NUCB2	WZSP003889	14	with SNP	non-synonymous SNP
X	POU3F4	WZSP016959	14	with SNP	
X	CYLC1	WZSP016958	14	with SNP	non-synonymous SNP
X	PLEKHA1	WZSP016957	14	with SNP	non-synonymous SNP

X	RPS6KA6	WZSP016956	14	with SNP	non-synonymous SNP
X	HMU	WZSP000677	14	with SNP	non-synonymous SNP
X	APOOL	WZSP000676	14	with SNP	non-synonymous SNP
X	SATL1	WZSP000675	14	with SNP	non-synonymous SNP
X	MEP1B	WZSP000674	14	with SNP	non-synonymous SNP
X	ZNF711	WZSP000673	14	with SNP	
X	POF1B	WZSP000672	14	with SNP	non-synonymous SNP
X	EIF5AL1	WZSP015028	14	with SNP	non-synonymous SNP
X	CHM	WZSP015027	14	with SNP	non-synonymous SNP
X	DACH2	WZSP015026	14	with SNP	
X	DACH2	WZSP015025	14	with SNP	non-synonymous SNP
X	DACH2	WZSP015024	14	with SNP	non-synonymous SNP
X		ENSSSCT00000017102	14		
X	DIRAS3	WZSP009281	14	with SNP	non-synonymous SNP
X	NA	WZSP009282	14	with SNP	non-synonymous SNP
X	NA	WZSP009283	14	with SNP	
X	KLHL4	WZSP009284	14	with SNP	non-synonymous SNP
X	UBE2D2	WZSP009285	14	with SNP	
X		ENSSSCT00000003677	14	with SNP	non-synonymous SNP
X	GAG	WZSP014386	14	with SNP	non-synonymous SNP
X	SMN1	WZSP018395	14	with SNP	non-synonymous SNP
X	HNRPDL	WZSP014748	14	with SNP	non-synonymous SNP
X	PCDH19	WZSP004391	18	with SNP	non-synonymous SNP
X	TNMD	WZSP004392	18		
X	TSPAN6	WZSP004393	18	with SNP	
X	SRPX2	WZSP004394	18	with SNP	non-synonymous SNP
X	SYTL4	WZSP004395	18	with SNP	non-synonymous SNP
X	NA	WZSP004396	17	with SNP	non-synonymous SNP
X	CSTF2	WZSP004397	17	with SNP	non-synonymous SNP
X	NOX1	WZSP004398	17	with SNP	non-synonymous SNP
X	XKRX	WZSP004399	17	with SNP	
X	ARL13A	WZSP004400	17	with SNP	non-synonymous SNP
X	TRMT2B	WZSP004401	17	with SNP	non-synonymous SNP
X	TMEM35	WZSP004402	17	with SNP	non-synonymous SNP
X	CENPI	WZSP004403	17	with SNP	non-synonymous SNP
X	DRP2	WZSP004404	17	with SNP	non-synonymous SNP
X	TAF7L	WZSP004405	17	with SNP	non-synonymous SNP
X	TIMM8A	WZSP004406	17	with SNP	non-synonymous SNP
X	BTK	WZSP004407	17	with SNP	
X	RPL36A	WZSP004408	17		
X	GLA	WZSP004409	17	with SNP	non-synonymous SNP
X	HNRNPH2	WZSP004410	17	with SNP	
X	ARMCX5	WZSP004411	17		
X	ARMCX1	WZSP004412	17	with SNP	
X	ARMCX6	WZSP004413	17	with SNP	non-synonymous SNP
X	FAM133A	WZSP004353	10	with SNP	non-synonymous SNP
X	NAP1L3	WZSP004354	9	with SNP	non-synonymous SNP
X		ENSSSCT00000027387	9		
X		ENSSSCT00000013670	9	with SNP	non-synonymous SNP
X	PCDH11X	WZSP017837	8	with SNP	non-synonymous SNP

Table S9. Functional gene categories enriched for genes on the LD block region.

Functional category	Pathway ID	*P* value*	Gene count
Shigellosis	ko05131	0.009	5 (157)
Basal transcription factors	ko03022	0.011	3 (57)
Neurotrophin signaling pathway	ko04722	0.016	6 (252)
Leukocyte transendothelial migration	ko04670	0.020	6 (263)
MAPK signaling pathway - fly	ko04013	0.041	2 (40)

*: *P* values represent *FDR*-adjusted *P* values.

Table S10. Overlapping QTLs with the LD block region on chromosome X.

QTL Class	QTL Type	Number	Percent (%)
Exterior	Behavioral	1	2.13
Meat & Carcass_Quality	Anatomy	10	78.72
	Fatness	23	
	Fat composition	3	
	Flavor	1	
Production	Growth	1	4.26
	Feed intake	1	
Reproduction	Reproductive organ	7	14.89
Total		47	100

Table S11. Indels exhibited significant difference between CnNorth and CnSouth.

Chr	Position	Gene ID	Gene Name	Ref	Mutations	*P* value[b]
X	47,631,511	WZSP015988	ITIH5L	T	TG	0.002
X	55,260,284	ENSSSCG00000012830	NA[a]	CTG	C	0.002
X	46,457,736	WZSP000020	HUWE1	ACT	A	0.003
X	55,260,561	ENSSSCG00000012830	NA	AG	A	0.003
X	55,260,563	ENSSSCG00000012830	NA	AG	A	0.004
9	2,830,288	WZSP017558	OLR226	T	TA	0.004

a: NA represents null.

b: *P* values represent *FDR*-adjusted *P* values.

Table S12. Top 100 SVs with different distribution (χ^2 with *FDR* correction, $P < 0.01$) between CnNorth and CnSouth.

Chr	SV_start	SV_end	χ^2 value	*P* value	CnSout	CnNouth	DS	BN	HX	LC	BM	WZSO	WZSI	DH	LT	GZT	HZT	NJ	MS	EHL	JH
X	56649999	56650381	11.12269	0.00085	9:00	0:06	0.00	0.00	0.00	0.00	0.00	0.00	0.00	0.00	0.00	95.29	82.46	86.91	73.30	89.27	77.75
X	56621130	56621617	11.12269	0.00085	9:00	0:06	0.00	0.00	0.00	0.00	0.00	0.00	0.00	0.00	0.00	89.73	96.51	66.32	85.63	87.27	66.53
X	52716780	52717188	11.12269	0.00085	9:00	0:06	0.00	0.00	0.00	0.00	0.00	0.00	0.00	0.00	0.00	76.96	82.60	86.76	87.25	75.74	83.09
X	51167855	51168221	11.12269	0.00085	9:00	0:06	0.00	0.00	0.00	0.00	0.00	0.00	0.00	0.00	0.00	78.96	78.69	73.77	90.71	80.05	75.96
X	50545147	50545558	11.12269	0.00085	9:00	0:06	0.00	0.00	0.00	0.00	0.00	0.00	0.00	0.00	0.00	76.16	56.93	59.85	78.83	80.78	76.89
X	46489272	46489621	11.12269	0.00085	9:00	0:06	0.00	0.00	0.00	0.00	0.00	0.00	0.00	0.00	0.00	78.51	81.66	81.66	80.80	90.26	77.36
8	28653516	28653919	11.12269	0.00085	9:00	0:06	0.00	0.00	0.00	0.00	0.00	0.00	0.00	0.00	0.00	90.32	90.07	97.77	83.13	90.32	88.59
7	36744448	36744742	11.12269	0.00085	9:00	0:06	0.00	0.00	0.00	0.00	0.00	0.00	0.00	0.00	0.00	68.71	94.22	94.56	69.39	75.85	68.03
6	9395723	9396080	11.12269	0.00085	9:00	0:06	0.00	0.00	0.00	0.00	0.00	0.00	0.00	0.00	0.00	84.87	85.99	85.71	89.08	87.11	88.24
6	104274314	104274831	11.12269	0.00085	9:00	0:06	0.00	0.00	0.00	0.00	0.00	0.00	0.00	0.00	0.00	83.17	82.98	66.34	61.32	58.41	69.63
4	6920252	6920684	11.12269	0.00085	9:00	0:06	0.00	0.00	0.00	0.00	0.00	0.00	0.00	0.00	0.00	82.87	98.38	74.07	71.53	77.31	81.25
3	134331933	134332355	11.12269	0.00085	9:00	0:06	0.00	0.00	0.00	0.00	0.00	0.00	0.00	0.00	0.00	67.06	68.96	65.40	54.74	66.59	68.72
2	76714858	76715420	11.12269	0.00085	9:00	0:06	0.00	0.00	0.00	0.00	0.00	0.00	0.00	0.00	0.00	91.64	88.79	93.06	88.26	92.53	91.28
2	104492528	104492842	11.12269	0.00085	9:00	0:06	0.00	0.00	0.00	0.00	0.00	0.00	0.00	0.00	0.00	75.80	64.97	75.48	69.43	75.48	77.39
13	158301585	158301965	11.12269	0.00085	9:00	0:06	0.00	0.00	0.00	0.00	0.00	0.00	0.00	0.00	0.00	92.63	97.37	88.42	92.11	90.26	88.42
X	61176608	61176932	8.136161	0.00434	8:01	0:06	0.00	0.00	0.00	0.00	78.09	0.00	0.00	0.00	0.00	71.91	69.14	70.06	60.49	72.53	64.20
9	35060416	35060942	8.136161	0.00434	8:01	0:06	0.00	0.00	0.00	95.63	0.00	0.00	0.00	0.00	0.00	78.71	92.78	78.33	96.20	79.28	82.32
9	1589156	1589591	8.136161	0.00434	8:01	0:06	0.00	85.06	0.00	0.00	0.00	0.00	0.00	0.00	0.00	84.83	80.46	88.74	87.82	79.31	86.44
9	105414999	105415410	8.136161	0.00434	8:01	0:06	0.00	83.94	0.00	0.00	0.00	0.00	0.00	0.00	0.00	100.00	94.89	94.65	86.86	92.94	91.00
7	64208088	64208405	8.136161	0.00434	8:01	0:06	0.00	0.00	0.00	0.00	0.00	0.00	0.00	83.60	0.00	84.54	85.49	91.80	75.39	82.33	85.17
6	137131771	137132264	8.136161	0.00434	8:01	0:06	0.00	96.15	0.00	0.00	0.00	0.00	0.00	0.00	0.00	94.32	96.15	94.32	93.10	93.91	95.33
5	53025976	53027811	8.136161	0.00434	8:01	0:06	0.00	98.20	0.00	0.00	0.00	0.00	0.00	0.00	0.00	96.35	97.00	99.29	94.66	97.22	96.35
4	27292849	27294729	8.136161	0.00434	8:01	0:06	0.00	0.00	0.00	0.00	0.00	0.00	0.00	93.99	0.00	94.57	93.19	93.24	93.51	92.98	93.09
3	99253194	99253582	8.136161	0.00434	8:01	0:06	0.00	0.00	0.00	0.00	0.00	0.00	0.00	0.00	84.54	74.74	77.06	59.54	79.64	81.44	75.77
3	83030825	83031138	8.136161	0.00434	8:01	0:06	0.00	91.69	0.00	0.00	0.00	0.00	0.00	0.00	0.00	89.78	87.22	94.89	73.80	94.25	86.90
3	57641287	57641727	8.136161	0.00434	8:01	0:06	94.09	0.00	0.00	0.00	0.00	0.00	0.00	0.00	0.00	91.82	93.41	91.36	56.14	90.68	89.77
3	130763540	130764080	8.136161	0.00434	8:01	0:06	0.00	0.00	0.00	0.00	0.00	0.00	0.00	0.00	66.11	67.41	38.33	91.67	60.00	38.33	91.30
2	58542798	58543128	8.136161	0.00434	8:01	0:06	0.00	93.33	0.00	0.00	0.00	0.00	0.00	0.00	0.00	86.97	88.48	86.36	87.27	90.91	85.76
2	129339767	129340307	8.136161	0.00434	8:01	0:06	0.00	0.00	0.00	0.00	87.59	0.00	0.00	0.00	0.00	83.15	83.70	83.89	86.67	86.48	85.19
15	75434127	75434652	8.136161	0.00434	8:01	0:06	0.00	0.00	46.86	0.00	0.00	0.00	0.00	0.00	0.00	55.05	55.24	49.52	41.52	89.52	47.24
15	126607677	126608074	8.136161	0.00434	8:01	0:06	0.00	0.00	0.00	0.00	0.00	0.00	0.00	0.00	63.22	64.74	76.32	69.77	60.96	64.99	65.99
14	78661588	78661846	8.136161	0.00434	8:01	0:06	0.00	0.00	0.00	0.00	89.53	0.00	0.00	0.00	0.00	88.37	88.37	93.80	63.57	87.21	96.90
14	55686663	55687047	8.136161	0.00434	8:01	0:06	0.00	0.00	0.00	0.00	0.00	0.00	0.00	81.25	0.00	68.23	71.88	66.41	60.42	70.57	70.83
1	41952346	41952759	8.136161	0.00434	8:01	0:06	77.97	0.00	0.00	0.00	0.00	0.00	0.00	0.00	0.00	57.38	79.90	58.11	91.53	54.48	92.98
13	170434068	170434455	8.136161	0.00434	8:01	0:06	0.00	0.00	0.00	0.00	95.87	0.00	0.00	0.00	0.00	89.92	87.08	89.66	66.41	93.02	89.15
13	142245359	142245666	8.136161	0.00434	8:01	0:06	0.00	0.00	0.00	0.00	93.16	0.00	0.00	0.00	0.00	67.10	75.57	74.27	52.77	73.29	70.68
13	113870939	113871273	8.136161	0.00434	8:01	0:06	0.00	0.00	0.00	0.00	88.62	0.00	0.00	0.00	0.00	97.31	94.61	88.62	92.51	90.42	90.42
1	251915698	251916093	8.136161	0.00434	8:01	0:06	0.00	88.35	0.00	0.00	0.00	0.00	0.00	0.00	0.00	93.16	89.62	94.68	69.62	93.92	85.32
1	25108244	25108801	8.136161	0.00434	8:01	0:06	0.00	97.13	0.00	0.00	0.00	0.00	0.00	0.00	0.00	92.64	90.13	94.61	84.38	99.46	97.13
1	23760029	23760451	8.136161	0.00434	8:01	0:06	0.00	79.15	0.00	0.00	0.00	0.00	0.00	0.00	0.00	90.52	82.70	80.81	85.78	75.83	52.61
12	31472476	31472888	8.136161	0.00434	8:01	0:06	0.00	94.90	0.00	0.00	0.00	0.00	0.00	0.00	0.00	93.20	95.87	94.66	54.37	96.12	93.20
1	224500628	224501031	8.136161	0.00434	8:01	0:06	0.00	64.76	0.00	0.00	0.00	0.00	0.00	0.00	0.00	93.05	88.83	66.75	58.56	68.98	89.33
1	221935912	221936528	8.136161	0.00434	8:01	0:06	0.00	96.43	0.00	0.00	0.00	0.00	0.00	0.00	0.00	93.34	93.67	94.48	95.78	95.45	97.89
1	18197224	18197586	8.136161	0.00434	8:01	0:06	0.00	85.91	0.00	0.00	0.00	0.00	0.00	0.00	0.00	73.48	92.82	72.38	66.85	74.59	76.80
1	160519011	160519408	8.136161	0.00434	8:01	0:06	0.00	0.00	89.67	0.00	0.00	0.00	0.00	0.00	0.00	94.46	97.48	92.19	60.20	88.92	79.09
1	15053219	15053650	8.136161	0.00434	8:01	0:06	0.00	0.00	0.00	0.00	0.00	0.00	0.00	100.00	0.0(	83.99	60.56	82.60	83.76	79.35	78.65
X	56812089	56812371	7.8125	0.00519	9:00	1:05	0.00	0.00	0.00	0.00	0.00	0.00	0.00	0.00	0.00	84.04	78.01	78.72	0.00	89.36	84.04
X	56587048	56587417	7.8125	0.00519	9:00	1:05	0.00	0.00	0.00	0.00	0.00	0.00	0.00	0.00	0.00	81.03	76.96	60.43	0.00	71.27	60.70
X	56256165	56256500	7.8125	0.00519	9:00	1:05	0.00	0.00	0.00	0.00	0.00	0.00	0.00	0.00	0.00	82.69	87.46	82.69	91.34	0.00	80.90
X	55844876	55847148	7.8125	0.00519	9:00	1:05	0.00	0.00	0.00	0.00	0.00	0.00	0.00	0.00	0.00	100.00	97.76	97.58	0.00	98.15	97.49
X	55718162	55718717	7.8125	0.00519	9:00	1:05	0.00	0.00	0.00	0.00	0.00	0.00	0.00	0.00	0.00	92.97	90.27	0.00	83.60	86.13	84.68
X	55396043	55396513	7.8125	0.00519	9:00	1:05	0.00	0.00	0.00	0.00	0.00	0.00	0.00	0.00	0.00	67.66	67.02	65.96	0.00	45.96	42.77
X	55373064	55373425	7.8125	0.00519	9:00	1:05	0.00	0.00	0.00	0.00	0.00	0.00	0.00	0.00	0.00	63.71	89.47	85.87	0.00	100.00	88.09
X	55180019	55180341	7.8125	0.00519	9:00	1:05	0.00	0.00	0.00	0.00	0.00	0.00	0.00	0.00	0.00	100.00	77.33	72.98	0.00	80.75	77.64
X	55122804	55123153	7.8125	0.00519	9:00	1:05	0.00	0.00	0.00	0.00	0.00	0.00	0.00	0.00	0.00	96.85	0.00	73.64	66.19	79.37	83.09
X	54897094	54897605	7.8125	0.00519	9:00	1:05	0.00	0.00	0.00	0.00	0.00	0.00	0.00	0.00	0.00	73.78	95.11	70.06	0.00	71.43	79.84
X	54884568	54885472	7.8125	0.00519	9:00	1:05	0.00	0.00	0.00	0.00	0.00	0.00	0.00	0.00	0.00	94.36	95.35	94.91	0.00	95.46	95.91
X	54745952	54746296	7.8125	0.00519	9:00	1:05	0.00	0.00	0.00	0.00	0.00	0.00	0.00	0.00	0.00	90.70	93.60	97.09	0.00	93.31	90.12
X	54626801	54627450	7.8125	0.00519	9:00	1:05	0.00	0.00	0.00	0.00	0.00	0.00	0.00	0.00	0.00	93.07	98.46	90.76	0.00	95.69	100.00
X	54310006	54313012	7.8125	0.00519	9:00	1:05	0.00	0.00	0.00	0.00	0.00	0.00	0.00	0.00	0.00	98.74	0.00	98.24	97.70	99.57	99.00
X	53438753	53443000	7.8125	0.00519	9:00	1:05	0.00	0.00	0.00	0.00	0.00	0.00	0.00	0.00	0.00	96.21	96.30	96.35	0.00	96.63	96.49
X	52568295	52568626	7.8125	0.00519	9:00	1:05	0.00	0.00	0.00	0.00	0.00	0.00	0.00	0.00	0.00	0.00	95.17	88.52	86.10	86.10	86.40
X	52126159	52128248	7.8125	0.00519	9:00	1:05	0.00	0.00	0.00	0.00	0.00	0.00	0.00	0.00	0.00	97.94	97.56	97.51	0.00	96.98	96.46
X	51505244	51506801	7.8125	0.00519	9:00	1:05	0.00	0.00	0.00	0.00	0.00	0.00	0.00	0.00	0.00	94.09	96.21	97.05	0.00	91.14	94.99
X	51294501	51294881	7.8125	0.00519	9:00	1:05	0.00	0.00	0.00	0.00	0.00	0.00	0.00	0.00	0.00	83.68	81.32	87.11	0.00	90.79	77.89
X	50841797	50842079	7.8125	0.00519	9:00	1:05	0.00	0.00	0.00	0.00	0.00	0.00	0.00	0.00	0.00	0.00	84.04	85.46	70.57	87.23	73.76
X	47628120	47628563	7.8125	0.00519	9:00	1:05	0.00	0.00	0.00	0.00	0.00	0.00	0.00	0.00	0.00	75.85	70.65	67.95	0.00	73.59	89.62
X	47456877	47457207	7.8125	0.00519	9:00	1:05	0.00	0.00	0.00	0.00	0.00	0.00	0.00	0.00	0.00	68.79	0.00	88.79	91.52	79.09	71.52
X	46916811	46917178	7.8125	0.00519	9:00	1:05	0.00	0.00	0.00	0.00	0.00	0.00	0.00	0.00	0.00	54.22	80.93	0.00	86.92	80.11	58.58
X	46875360	46875779	7.8125	0.00519	9:00	1:05	0.00	0.00	0.00	0.00	0.00	0.00	0.00	0.00	0.00	66.59	84.73	83.53	0.00	81.62	70.41
X	46870053	46870408	7.8125	0.00519	9:00	1:05	0.00	0.00	0.00	0.00	0.00	0.00	0.00	0.00	0.00	0.00	89.58	89.58	89.86	90.70	88.45
X	46797688	46798016	7.8125	0.00519	9:00	1:05	0.00	0.00	0.00	0.00	0.00	0.00	0.00	0.00	0.00	81.10	68.90	89.33	0.00	72.56	89.63
X	22061209	22068673	7.8125	0.00519	9:00	1:05	0.00	0.00	0.00	0.00	0.00	0.00	0.00	0.00	0.00	98.14	0.00	93.70	98.06	98.86	98.71

9	84678853	84679776	7.8125	0.00519	9:00	1:05	0.00	0.00	0.00	0.00	0.00	0.00	0.00	0.00	0.00	0.00	96.53	90.03	91.33	95.77	96.75
9	47192312	47192669	7.8125	0.00519	9:00	1:05	0.00	0.00	0.00	0.00	0.00	0.00	0.00	0.00	0.00	64.15	93.00	93.84	0.00	99.72	60.78
9	119181432	119181865	7.8125	0.00519	9:00	1:05	0.00	0.00	0.00	0.00	0.00	0.00	0.00	0.00	0.00	89.38	89.15	81.52	0.00	97.00	90.99
9	118464432	118464844	7.8125	0.00519	9:00	1:05	0.00	0.00	0.00	0.00	0.00	0.00	0.00	0.00	0.00	0.00	61.65	66.50	57.52	64.56	63.11
9	106867921	106868263	7.8125	0.00519	9:00	1:05	0.00	0.00	0.00	0.00	0.00	0.00	0.00	0.00	0.00	100.00	82.46	73.98	0.00	78.65	77.49
8	44813238	44815237	7.8125	0.00519	9:00	1:05	0.00	0.00	0.00	0.00	0.00	0.00	0.00	0.00	0.00	97.00	99.45	96.70	0.00	96.80	97.50
8	34019994	34020324	7.8125	0.00519	9:00	1:05	0.00	0.00	0.00	0.00	0.00	0.00	0.00	0.00	0.00	83.94	91.21	90.00	46.06	0.00	96.97
8	126480769	126481230	7.8125	0.00519	9:00	1:05	0.00	0.00	0.00	0.00	0.00	0.00	0.00	0.00	0.00	86.98	0.00	97.83	41.21	82.00	90.02
8	126171717	126172250	7.8125	0.00519	9:00	1:05	0.00	0.00	0.00	0.00	0.00	0.00	0.00	0.00	0.00	75.42	58.16	73.73	0.00	66.79	60.98
7	73240504	73240826	7.8125	0.00519	9:00	1:05	0.00	0.00	0.00	0.00	0.00	0.00	0.00	0.00	0.00	97.83	0.00	87.89	83.85	86.34	84.78
6	39843345	39843697	7.8125	0.00519	9:00	1:05	0.00	0.00	0.00	0.00	0.00	0.00	0.00	0.00	0.00	82.67	84.66	92.90	0.00	92.33	80.97
6	39840092	39840637	7.8125	0.00519	9:00	1:05	0.00	0.00	0.00	0.00	0.00	0.00	0.00	0.00	0.00	91.19	93.21	70.09	0.00	43.12	88.07
6	143732994	143733247	7.8125	0.00519	9:00	1:05	0.00	0.00	0.00	0.00	0.00	0.00	0.00	0.00	0.00	92.49	96.84	0.00	87.35	93.68	88.54
6	143469491	143472678	7.8125	0.00519	9:00	1:05	0.00	0.00	0.00	0.00	0.00	0.00	0.00	0.00	0.00	97.08	0.00	96.80	97.08	96.80	96.52
5	64860026	64860520	7.8125	0.00519	9:00	1:05	0.00	0.00	0.00	0.00	0.00	0.00	0.00	0.00	0.00	74.70	90.89	96.36	84.21	76.11	0.00
5	44706640	44707034	7.8125	0.00519	9:00	1:05	0.00	0.00	0.00	0.00	0.00	0.00	0.00	0.00	0.00	90.86	88.83	86.29	0.00	87.82	85.53
5	14511259	14511692	7.8125	0.00519	9:00	1:05	0.00	0.00	0.00	0.00	0.00	0.00	0.00	0.00	0.00	96.54	83.37	85.91	0.00	86.84	87.30
5	105376621	105376951	7.8125	0.00519	9:00	1:05	0.00	0.00	0.00	0.00	0.00	0.00	0.00	0.00	0.00	89.09	82.42	0.00	80.91	85.45	91.52
5	100630499	100630913	7.8125	0.00519	9:00	1:05	0.00	0.00	0.00	0.00	0.00	0.00	0.00	0.00	0.00	0.00	53.14	77.05	85.02	77.05	56.04
4	95747330	95747738	7.8125	0.00519	9:00	1:05	0.00	0.00	0.00	0.00	0.00	0.00	0.00	0.00	0.00	63.97	67.89	92.65	0.00	94.36	87.99
4	85470869	85471174	7.8125	0.00519	9:00	1:05	0.00	0.00	0.00	0.00	0.00	0.00	0.00	0.00	0.00	92.79	90.49	83.61	0.00	83.28	83.61
4	80049873	80050278	7.8125	0.00519	9:00	1:05	0.00	0.00	0.00	0.00	0.00	0.00	0.00	0.00	0.00	74.57	88.40	89.88	0.00	74.07	80.49
4	74875341	74875732	7.8125	0.00519	9:00	1:05	0.00	0.00	0.00	0.00	0.00	0.00	0.00	0.00	0.00	98.21	80.31	83.12	0.00	84.40	91.05
4	48650460	48650931	7.8125	0.00519	9:00	1:05	0.00	0.00	0.00	0.00	0.00	0.00	0.00	0.00	0.00	90.66	88.32	92.14	95.33	92.36	0.00
4	33444329	33444678	7.8125	0.00519	9:00	1:05	0.00	0.00	0.00	0.00	0.00	0.00	0.00	0.00	0.00	83.09	80.80	0.00	84.81	85.96	90.83
4	33012792	33013199	7.8125	0.00519	9:00	1:05	0.00	0.00	0.00	0.00	0.00	0.00	0.00	0.00	0.00	98.77	0.00	92.38	86.98	90.66	81.57
4	31169895	31170923	7.8125	0.00519	9:00	1:05	0.00	0.00	0.00	0.00	0.00	0.00	0.00	0.00	0.00	97.86	96.11	97.47	95.33	0.00	95.23

Table S13. SVs exhibited significant difference between CnNorth and CnSouth.

Chr	SV_Start	SV_End	Gene ID	Gene Name	*P* value[b]
X	56,649,999	56,650,381	WZSP004769	EDA	0.000853
X	56,621,130	56,621,617	WZSP004769	EDA	0.000853
X	52,716,780	52,717,188	WZSP003802	HEPH	0.000853
X	51,167,855	51,168,221	NA[a]	NA	0.000853
X	50,545,147	50,545,558	WZSP013116	ARHGEF9	0.000853
X	46,489,272	46,489,621	WZSP000020	HUWE1	0.000853

a: NA represents null.

b: *P* values represent *FDR*-adjusted *P* values.

Table S14. Adult body sizes from small and large pigs in this study.

Body size	Small pigs (N = 7)						
(cm)	WZSI[#]	WZSO[*]	BN[#]	BM[*]	HX[*]	HZT[*]	GZT[#]
length	53.86±0.40	69.55±2.02	89.02±1.84	81.73±0.87	92.78±0.62	76.00	94.56±1.25
height	32.06±0.62	48.13±1.19	41.08±1.34	42.68±0.53	47.44±0.29	44.66	48.32±0.89
Body size	Large pigs (N = 7)						
(cm)	DP[*]	LP[*]	LW[*]	PP[*]	HP[*]	NJ[*]	MP[*]
length	161.70±0.77	165.00±2.47	160.10±1.73	157.40±1.08	121.50±1.60	156.78±4.76	152.20±0.81
height	94.67±0.66	89.40±0.97	90.30±0.65	86.80±1.02	69.33±0.95	82.88±4.07	89.10±0.71

Notes: Body sizes of adult male are represented in the table.

(1) * Data of body sizes were available for the book *Animal genetic resources in China: pigs*.

(2) # Data of body sizes were obtained from measurements. See Materials and Methods for more details of measurements.

Table S15. Candidate genes detected in accordance with >80% in one group and <20% in the other.

Gene name	KLHL2、PARP4、USP8、CEP152、 C7ORF41、 ORC1L、TMEM48、R3HDM2、C1ORF173、SPATS2、MCRS1、FAM186B、TPT1、PLA2G6、TRIOBP、OR2W6P、ERP27、KALRN、RP1、TADA3L、TIMP4、RP2、SHROOM4、KIAA0564、GPR179、ARHGAP23、UNC13B、ZNF536、KIAA1632、EEF1A1、FBXW8、DHX37、CHUK、PKD2L1、CLN5、RWDD2B、TP53BP2、DSCAML1、NOL9、CALD1、TBXAS1、EDC4、NRN1L、RASSF8、CEP290、MRPL42、PLXNC1、FER1L5、RTN1、CCDC46、SLC35D3、DYSF、CCDC129、FRMPD4、SCML2、OBSCN、FZD10、ABCC12、NARS2、ALPK2、IGSF5、HTR1F、DCHS2、CYP7A1、UBXN2B、TG、OR11L1、PPP1R2、AK3L1、POP1、RBM44、OR52B4、TAS2R42、IYD、SYNE1、ARID1B、C9ORF50、ADAMTS20、PUS7L、NELL2 、ENSSSCT00000013458 、 ENSSSCT00000022574 、 ENSSSCT00000034690 、WZSP006486、WZSP006492、WZSP008589、WZSP009073、WZSP009675、SLC34A1、OR11A1、LEPR、ABCB5、FBXL13、FANCC、WZSP010732

Table S16. Indels exhibited significant difference between pig populations of big size and small size.

Chr	Position	Gene ID	Gene Name	Ref	Mutations	*P* value[b]
18	39,195,808	WZSP014727	POL	C	CT	0.001
X	57,710,290	WZSP003278	NA	CA	C	0.002
1	277,774,937	WZSP008322	COL1A1	G	GCACC	0.007
13	113,947,040	WZSP000099	NA[a]	TG	T	0.007
1	46,519,739	WZSP008146	KRTAP13-3	A	AT	0.007
4	71,908,134	WZSP015432	ASPH	T	TAC	0.011
14	127,978,228	WZSP015654	HSPA12A	GT	G	0.012
14	31,174,192	ENSSSCT00000010754	NA	TCA	T	0.012
X	44,129,462	ENSSSCT00000013458	NA	GA	G	0.012
13	65,877,573	WZSP004887	C3ORF32	C	CG	0.019

a: NA represents null.

b: *P* values represent *Bonferroni*-adjusted *P* values.

Table S17. SVs exhibited significant difference between pig populations of big size and small size.

Chr	SV_Start	SV_End	Gene ID	Gene Name	*P* value[b]
7	93,247,772	93,248,158	WZSP001164	ZFYVE9	0.001
1	168,985,923	168,988,954	WZSP019179	PAQR5	0.007
11	71,782,155	71,782,585	WZSP002550	PCCA	0.007
1	234,672,716	234,673,097	NA[a]	NA	0.007
13	203,507,887	203,508,305	WZSP014216	HLCS	0.007
13	84,648,052	84,650,439	NA	NA	0.007
18	9,552,906	9,553,376	WZSP005333	SLC37A3	0.007
3	61,438,906	61,443,124	NA	NA	0.007
4	130,045,160	130,045,517	NA	NA	0.007
7	2,220,680	2,221,123	NA	NA	0.007
10	51,097,752	51,098,188	WZSP008493	MASTL	0.026
10	74,060,780	74,061,248	NA	NA	0.026
1	108,637,313	108,638,042	WZSP016945	PLEKHO2	0.026
1	110,112,629	110,112,962	NA	NA	0.026
11	13,938,516	13,938,967	NA	NA	0.026
11	24,677,970	24,678,417	NA	NA	0.026
1	13,259,159	13,259,981	NA	NA	0.026
1	141,591,823	141,592,130	NA	NA	0.026
11	4,519,009	4,519,382	NA	NA	0.026
1	151,691,370	151,691,735	NA	NA	0.026

a: NA represents null.

b: *P* values represent *Bonferroni*-adjusted *P* values.
